# Environmental pollution and extreme weather conditions: insights into the effect on mental health

**DOI:** 10.3389/fpsyt.2024.1389051

**Published:** 2024-05-28

**Authors:** Maciej Tota, Julia Karska, Szymon Kowalski, Natalia Piątek, Magdalena Pszczołowska, Katarzyna Mazur, Patryk Piotrowski

**Affiliations:** ^1^ Faculty of Medicine, Wroclaw Medical University, Wroclaw, Poland; ^2^ Department of Psychiatry, Wroclaw Medical University, Wroclaw, Poland

**Keywords:** environmental pollution, air pollution, anxiety, schizophrenia, depression, extreme weather conditions

## Abstract

Environmental pollution exposures, including air, soil, water, light, and noise pollution, are critical issues that may implicate adverse mental health outcomes. Extreme weather conditions, such as hurricanes, floods, wildfires, and droughts, may also cause long-term severe concerns. However, the knowledge about possible psychiatric disorders associated with these exposures is currently not well disseminated. In this review, we aim to summarize the current knowledge on the impact of environmental pollution and extreme weather conditions on mental health, focusing on anxiety spectrum disorders, autism spectrum disorders, schizophrenia, and depression. In air pollution studies, increased concentrations of PM2.5, NO2, and SO2 were the most strongly associated with the exacerbation of anxiety, schizophrenia, and depression symptoms. We provide an overview of the suggested underlying pathomechanisms involved. We highlight that the pathogenesis of environmental pollution-related diseases is multifactorial, including increased oxidative stress, systematic inflammation, disruption of the blood-brain barrier, and epigenetic dysregulation. Light pollution and noise pollution were correlated with an increased risk of neurodegenerative disorders, particularly Alzheimer’s disease. Moreover, the impact of soil and water pollution is discussed. Such compounds as crude oil, heavy metals, natural gas, agro-chemicals (pesticides, herbicides, and fertilizers), polycyclic or polynuclear aromatic hydrocarbons (PAH), solvents, lead (Pb), and asbestos were associated with detrimental impact on mental health. Extreme weather conditions were linked to depression and anxiety spectrum disorders, namely PTSD. Several policy recommendations and awareness campaigns should be implemented, advocating for the advancement of high-quality urbanization, the mitigation of environmental pollution, and, consequently, the enhancement of residents’ mental health.

## Graphical Abstract

Suggested impact of environmental pollution in the pathogenesis of mental disorders. ICAM-1, intercellular cell adhesion molecule-1; VCAM-1, vascular cell adhesion molecule-1; DCF, density compensation function; CBF, cerebrospinal fluid; ROS, reactive oxygen species.

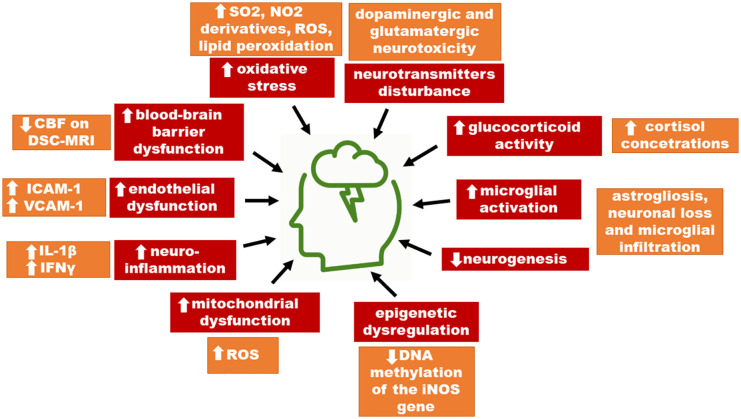

## 1 Introduction

Environmental pollution presents a global challenge, affecting numerous aspects of public health. Growing evidence suggests a correlation between various pollutants such as air, soil, water, light, and noise pollution, as well as extreme weather conditions (hurricanes, floods, wildfires, and droughts), and the development of mental disorders. These pollutants can influence the human nervous system, resulting in notable effects on mental health. They can alter neural tissue function and gene expression and also potentially cause social stress linked to environmental degradation. Within this framework, environmental pollution acts as a psychogenic factor, triggering psychological trauma and disrupting individual homeostasis (1).

Anxiety disorders are the most common psychiatric conditions that significantly worsen the quality of life. According to large population studies, the lifetime prevalence is estimated at 30-35% in the United States (2–4). Anxiety is a broad term that comprises many subtypes, namely, panic attacks, post-traumatic stress disorder (PTSD), generalized anxiety disorder (GAD), phobias, obsessive-compulsive disorder (OCD), and social anxiety disorder (SAD) (5).

Schizophrenia is a mental disorder marked by hallucinations, disorganized speech, and delusions. The condition, which affects 1% of the world’s population, is one of the leading 10 global causes of disability (6). Despite the estimated 60-80% heritability of schizophrenia, it is highly believed that several environmental factors contribute to the emergence (7–9). Of these, maternal infections and obstetric complications during pregnancy, winter or spring birth, childhood adversity, cannabis use, and urban living are frequently linked to an elevated incidence of schizophrenia (6, 10).

Autism spectrum disorders (ASDs) are a heterogeneous group of neurodevelopmental disorders. The etiology of ASDs remains inconclusive, but research suggests genetic, epigenetic, and environmental contributing factors and likely prenatal origins (11–13). Its genetic heterogeneity is remarkable – more than 800 ASD predisposition genes are identified. Experimental findings indicate that there are commonalities in the pathways affected by both genetic mutations and environmental factors. This suggests that there are intersections and intricate interactions between genetic vulnerability and harmful substances like air pollutants in their impact on ASD. Research involving animal models exposed to air pollutants has revealed a variety of intricate impacts on the central nervous system (CNS). The underlying mechanisms often involve oxidative stress and neuroinflammation (11).

Depressive disorders affect approximately 5-6% of the population and can lead to an increased risk of suicide. Moreover, depression is also associated with a higher risk of morbidity and mortality from cardiovascular and respiratory diseases, and vice versa somatic conditions may be aggravating factors that worsen depression symptoms (14–16).

Despite many hypotheses, the pathomechanisms of environmental pollution-related diseases have not been fully elucidated yet. Our review provides a comprehensive overview of the current understanding of the relationship between environmental pollution and the emergence of mental health, including the most common disorders, e.g., anxiety disorders, schizophrenia, autism spectrum disorder and depression.

## 2 Materials and methods

**Figure 1:**
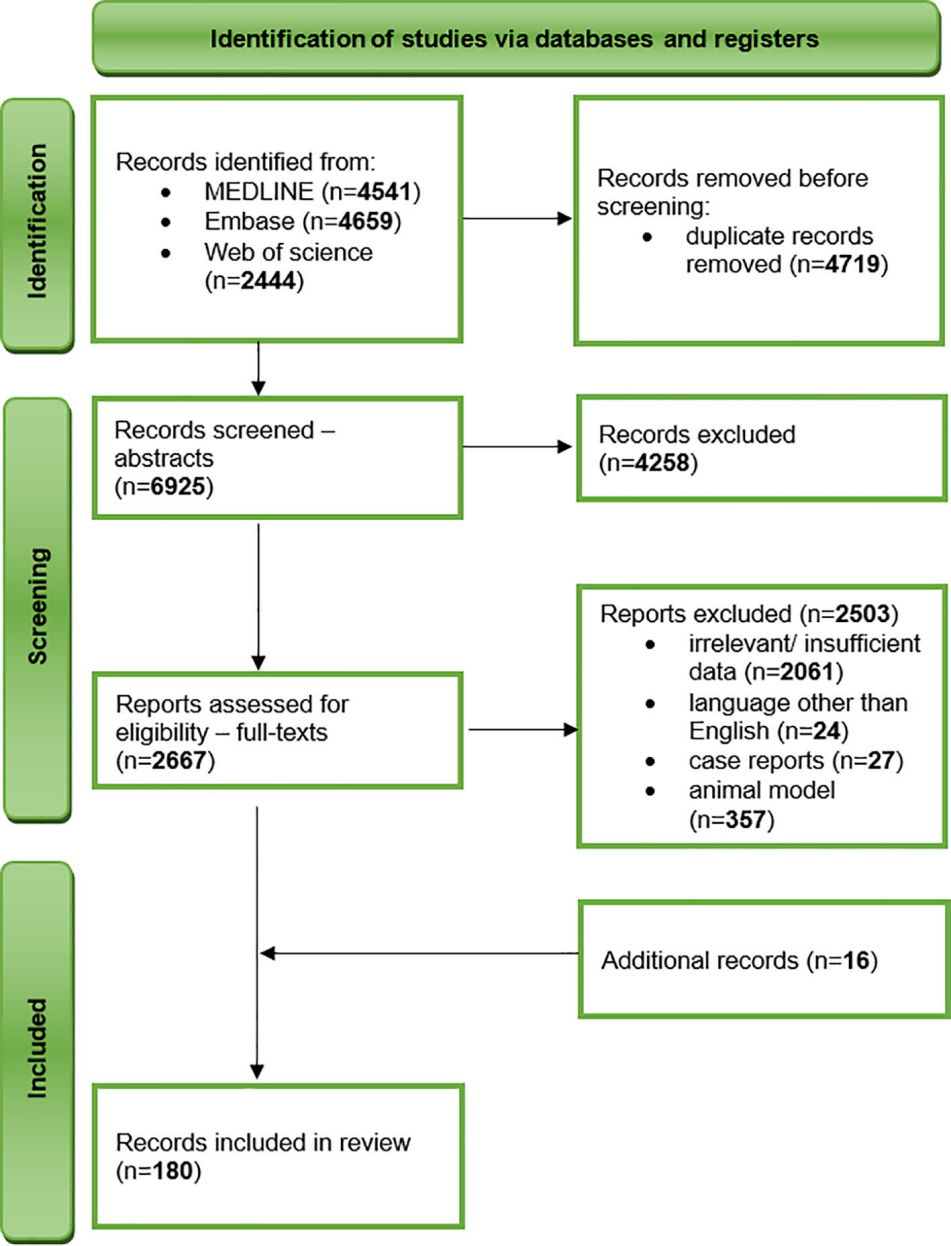
A flow diagram illustrating the process of literature research.

We undertook a comprehensive narrative review of literature published before 10 November 2023 in three databases – MEDLINE, Embase, and Web of Science. After an initial literature research by two independent researchers, we developed a search strategy including the following keywords: “air pollution” and “schizophrenia”, “air pollution” and “anxiety”, “air pollution” and “depression”, “air pollution” and “autism”, “noise pollution” and “depression”, “noise pollution” and “anxiety”, “heavy metals” and “depression”, “heavy metals” and “anxiety”, “fertilisers” and “depression”, “fertilisers” and “anxiety”, “light pollution” and “depression”, “light pollution” and “anxiety”. The inclusion criteria included original research and review articles, studies on humans, and the English language. The exclusion criteria comprised insufficient or irrelevant data, conference materials, book chapters, letters to editors, case reports, animal and *in vitro* models, and language other than English. To ensure the completeness of data collection, we have also performed a manual evaluation of reference lists of included studies (**Figure 1**).

## 3 Air pollution

Ambient air pollution (AAP), including ambient particulate matter (PM2.5, PM10), ozone (O3), nitrogen dioxide (NO2), sulfur dioxide (SO2), and carbon monoxide (CO), is associated with detrimental health outcomes including respiratory and cardiovascular disorders, as well as increased premature mortality risk (17–21). PM2.5 is defined as particulate matter with a diameter of 2.5 μm or less, while PM10 refers to particles with a diameter of 10 μm or less. Large conducting airways are where PM10 is deposited. Meanwhile, PM2.5 can overcome the alveolar-capillary barrier and reach other organs. A common hallmark in the pathophysiology of many of these disorders linked to exposure to various airborne pollutants is inflammation (22). Several studies suggested that exposure to AAP may affect mental health, and those are herein reviewed and summarized.

The air pollutants may enter the CNS in two different ways. The first one is a direct way via the olfactory system. Nasal inhalation allows the smaller particles to contact with olfactor receptors or trigeminal nerve, by which air pollutants are transported to the brain. The second way is systemic inflammation. The air pollutants that reach the alveolars during breathing, provoke inflammation in lung tissue. As a consequence, particles are transported to the systemic circulation and subsequently pass through the blood-brain barrier (23). After crossing the blood-brain barrier, air pollutants may contribute to the development of mental disorders. The exact mechanism is complex and likely depends on interactions with other risk factors. However, neuroinflammation and oxidative stress play an important role. Air pollutants are factors that cause proinflammatory mediators to release. Chronic respiratory and systemic inflammation affects the brain and induces neuroinflammation and an increased production of reactive oxygen species (ROS). Additionally, the particles stimulate specific mechano-receptors, which leads to the lung arc reflex. Likewise, through the stimulation of mechano-receptors the sympathetic nervous system is activated and vasoconstrictors released. Furthermore, air pollutants contribute to the direct formation of ROS that damage the blood-brain barrier and alter its permeability (23, 24).

### 3.1 Air pollution and anxiety disorders

Zhou et al. found a positive association between SO2 and NO2 pollutants and increased outpatient anxiety visits (4.11% and 3.97% increase, respectively). No differences between gender and age were observed. Although PM10 was not substantially correlated with anxiety-related outpatient visits in the single-pollutant model, it could be a confounding factor that increased anxiety-related adverse effects of SO2 and NO2. Authors suggest that may be an outcome of a synergistic effect of pollutant combinations (5).

Hautekiet et al. evaluated the correlation between long-term annual exposure to PM2.5, black carbon (BC), NO2, and self-rated health. It was noted that higher exposure to PM2.5 and BC resulted in an increased risk of GAD. All three examined pollutants were positively associated with poor self-rated health (**Table 1**). This study showed neither an association between long-term AAP and severe psychological distress or suicidal behavior. Interestingly, after including physical activity levels in the analyses, the correlations between air pollution and mental or self-rated health failed to reach statistical significance (25). Thus, the benefits of exercise for mental health may outweigh the detrimental effects of air pollution.

**Table 1:** Overview of studies on air pollution and anxiety disorders among various populations.

Study group	Year of publication	Country	N° of patients	End-point/scale assessed	Proposed mechanism/hypothesis	Time of exposure	Influence on disease incidence	Ref.
general population	2021	China	23,773	N° of outpatient visits	↑oxidative stress – ROS generated by SO2 derivatives and NO2 ↑mitochondrial morphological changes ➔ reduced ATP production and decreased respiratory complexes by NO2	short-term	10 μg/m3 increase corresponding to the increase of outpatient anxiety visits at 4.11% (95% CI: 2.15%, 6.06%) for SO2 and 3.97% (95% CI: 1.90%, 6.06%) for NO2	(5)
>15 years old	2022	Belgium	16,455	General Health Questionnaire (GHQ-12), Symptom Checklist-90-Revised (SCL-90-R), Short Form Health Survey (SF-36)	↑inflammation	long-term	3.8 µg/m3 increase in long-term PM2.5 (IQR) was associated with increased odds of suboptimal vitality (OR=1.27; 95% CI: 1.13, 1.42), poor self-rated health (OR=1.20; 95% CI: 1.09, 1.32) and depressive disorder (OR=1.19; 95% CI: 1.00, 1.41); 10.8 µg/m3 increment in NO2 exposure increases the odds of psychological distress (OR=1.06; 95% CI: 0.99,1.14), suboptimal vitality (OR=1.13; 95% CI: 1.03, 1.23) and poor self-rated health (OR=1.14 95% CI; 1.06, 1.22)	(25)
general population, 56 cities	2022	China	57,124	N° of daily hospital admissions	↑inflammation ↑oxidative stress	short-term	10 μg/m3 increases in NO2 at lag0 and SO2 at lag6 were associated with significant increases of 1.37% (95% CI: 0.14%, 2.62%) and 1.53% (95% CI: 0.59%, 2.48%) in hospital anxiety admissions, respectively	(26)
women aged 57-85 (mean 70) years	2015	United States	71,271	the subscale of the Crown-Crisp index	↑inflammation ↑oxidative stress	short-term	OR per 10 µg/m3 increase in prior one-month average PM2.5: 1.12, 95% CI 1.06 to 1.19; in prior 12 month average PM2.5: 1.15, 1.06 to 1.26)	(27)
57–85 years	2017	United States	4,008	Hospital Anxiety and Depression Scale (HADS)	↑inflammation ↑oxidative stress ↑markers of glucocorticoid activity ↑levels of the stress hormone cortisol ↑PM-mediated aggravation of cardiopulmonary conditions	long-term	PM2.5 was significantly associated with anxiety symptoms, with the most significant increase for a 180-day moving average (OR = 1.61; 95% CI: 1.35, 1.92) after adjusting for socioeconomic measures	(28)
outdoor employees	2022	Pakistan	299	four-item scale developed by Abramis (1994) (29)	ND	ND	at 95% CI, air pollution had a significant negative association with employees’ performance (β+0.173, p<0.018); in the presence of anxiety, air pollution more severely affects the employees’ performance	(30)
general population	2022	China	101,636	N° of outpatient visits	↑inflammation ↑oxidative stress	short-term	each 10 μg/m3 increase of PM2.5, PM10, NO2, and SO2 was associated with a 1.51% (95% CI: 0.61%, 2.43%), 1.58% (95% CI: 0.89%, 2.28%), 13.95% (9.98%, 18.05%) and 11.84% (95% CI: 8.25%, 15.55%) increase in outpatient anxiety visits	(31)
pregnant women	2021	South Korea	1,481	Korean version of the State-Trait Anxiety Inventory (K-STAI)	↑inflammation ↑oxidative stress dysregulation of the endocrine system or metabolic processes; disturbance of neurotransmitters	short-term	exposure to PM2.5, PM10, and NO2 during the second trimester was significantly associated with anxiety symptoms	(32)
children (mean12.2 years old)	2019	United States	344	Spence Children’s Anxiety Scale (SCAS)	↑inflammation ↑oxidative stress ↑dopaminergic and glutamatergic neurotoxicity; altered synaptic plasticity	long-term	exposure to ECAT at birth was associated with increased child-reported anxiety; each 0.25 μg/m3 increase in ECAT was associated with a 2.3 point increase (95% CI 0.8–3.9) in SCAS total anxiety score	(33)
adults (45-74 years old)	2017	Spain	958	self-reported history of anxiety and depression disorders and self-reported medication use	↑inflammation ↑oxidative stress	long-term	correlations regarding anxiety disorders did not reach statistical significance - increased odds of anxiety of 1.49 (95% CI; 0.62, 3.58) for each 5 µg/m3 PM2.5 increase, 1.33 (95% CI 0.48, 3.68) for each 10 µg/m3 PM10 increase; 1.27 (95% CI 0.91, 1.77) for each 20 µg/m3 NO2 increase	(34)
children	2019	8 European countries	13,182	Child Behaviour Checklist for ages 6–18 (CBCL/6-18)	↑inflammation ↑oxidative stress ↑neurodegeneration	long-term	no association between prenatal and postnatal exposure to air pollution with depressive and anxiety symptoms and aggressive behaviour in children between 7 and 11 years old	(35)
men (21-80 years old)	2012	United States	735	Brief Symptom Inventory (BSI)	↓DNA methylation of the iNOS gene	short-term	participants with high anxiety scores had a decrease in iNOS methylation 3 times greater than participants with low anxiety; participants with low optimism scores had a decrease in iNOS methylation 4 times greater than participants with high optimism	(36)

ROS, reactive oxygen species; IQR, interquartile range; CI, confidence interval; OR, odds ratio; ATP, adenosine triphosphate; ND, no data; iNOS, inducible nitric oxide synthase, ECAT, elemental carbon attributed to traffic.

Power et al. assessed the influence of PM2.5 and PM10 on anxiety symptoms in women. The authors demonstrated that increased short-term PM2.5 exposure contributed to a significant rise in anxiety prevalence. No such correlation was confirmed for PM10, and there was no dose-dependent relationship between anxiety symptoms in households near major roadways (27).

The NSHAP (National Social Life, Health and Aging Project) study has also confirmed the association between PM2.5 and anxiety. Individuals with low socioeconomic status and comorbidities were more likely to exhibit identified relationships. Notably, shorter-term exposure was more relevant to the development of anxiety symptoms (28).

Bari et al. suggested that air pollution may negatively impact employees’ performance. Authors point out that anxiety is the mediator in that correlation (30). Nonetheless, the sample size was relatively small (299 participants) and comprised only pharmaceutical workers. Hence, future studies are needed to corroborate those findings.

Another multicity study in China evaluated the role of PM2.5, PM10, SO2, and NO2. Additionally, twelve cold spell definitions were evaluated. Authors found that cold spells and air pollution are significant contributors to anxiety, and exposure to these two together may have synergistic effects on anxiety (31).

Pregnancy makes women particularly vulnerable to many diseases. Lamichhane et al. evaluated whether air pollution predisposes to anxiety and depression in that group. Exposure to PM2.5, PM10, and NO2 during the second trimester was associated with anxiety symptoms and O3 exposal during the third trimester correlated with an increased risk of depressive symptoms. Notably, stronger correlations between PM2.5 and PM10 with anxiety symptoms were found among participants with a history of smoking (32).

Another group that may be more susceptible to air pollution is the child population (37). Elemental Carbon Attributable to Traffic (ECAT), a substitute for diesel exhaust, was measured at birth, at age 12, and during average exposure throughout childhood. Interestingly, the results show that early childhood exposure constituted the most relevant period of exposure regarding the outcomes of depression and was also significantly associated with anxiety symptoms (33). Similarly, Peterson et al. suggest that prenatal exposure to PM2.5 and polycyclic aromatic hydrocarbons (PAH) may be associated with an increased risk of anxiety and attention-deficit/hyperactivity disorder (ADHD) in youth. It is suggested that inflammation and oxidative stress could disrupt brain development (38). Notwithstanding, Jorcano et al. could not confirm this association. The authors did not observe a correlation between prenatal and postnatal exposure to air pollution with depressive, anxiety, and aggressive symptoms in children between 7 and 11 years old (35). Consequently, the lack of associations in that paper may indicate that the study population is too young to have developed emotional and behavioural issues linked to exposure to air pollution. We suggest that symptoms could be more likely to manifest later in life.

Vert et al. examined the association between air pollution and a history of anxiety and depression, as well as medication use (benzodiazepines and antidepressants). The study showed that long-term exposure to air pollution may raise the risk of depression, as well as increase antidepressant and benzodiazepine intake. Although anxiety disorder associations were insufficient to reach statistical significance, odds ratios were higher than 1 (34) (**Table 1**).

Altered DNA methylation is hypothesized as one of the putative mechanisms linking air pollution exposure to anxiety. Madrigano et al. assessed the association between DNA methylation of *iNOS* (inducible nitric oxide synthase) genes with PM2.5 and black carbon (BC). Notably, *iNOS* methylation was decreased after acute exposure to both BC and PM2.5. A 1 μg/m3 increase in exposure to BC 4 hours prior to the clinical test was linked to a 0.9% decrease in 5-methylcytosine in *iNOS*. A 10 μg/m3 elevation in exposure to PM2.5 was associated with a 0.6% reduction in 5-methylcytosine in *iNOS*. The *iNOS* methylation was decreased by three times in people with high anxiety scores compared to participants with low anxiety scores. Individuals with low optimism levels experienced a four times reduction in *iNOS* methylation than participants with high optimism scores (36) (**Table 1**).

### 3.2 Air pollution and schizophrenia

Ji et al. investigated the effect of short-term exposure to O3, PM2.5, PM10, NO2, SO2, and CO on hospitalization for schizophrenia. They found that PM2.5, PM10, SO2, and CO concentrations were associated with an increased number of hospital admissions. SO2 pollutant, in particular, was strongly associated with the examined endpoint. Females and individuals under age 45 were more susceptible to air pollution (39).

Another study found that the risk of hospital admission for schizophrenia depending on PM2.5 or PM10 waves correlates with marital status and sex – a higher risk was observed among married and female patients. PM2.5 wave was defined as ≥3 consecutive days with PM2.5 concentration ≥90th, ≥92.5th, ≥95th, and ≥97.5th percentile. Such air conditions corresponded to 5.0% (2.3%–7.8%), 5.1% (1.9%–8.4%), 6.9% (3.0%–10.8%) and 12.0% (5.3%–19.1%) risk of schizophrenia hospitalization, respectively (40). One possible explanation for the higher incidence of schizophrenia hospital admissions among married patients is that they may receive more prompt and appropriate attention from their spouses.

Hospital re-admission due to schizophrenia exacerbations is another issue that was investigated in the concern of air pollution (41). Both PM2.5 and PM10 concentrations were positively correlated with hospital re-admissions. Younger patients (<45 years old) were more susceptible, consistent with other studies. Although, unlike in other studies – stronger associations were found in males (41).

A nationwide study conducted in the United States found that the effect of PM2.5 on hospital schizophrenia admissions is not immediate after exposure but appears 3-6 days later (42). It suggests the delayed type of response, yet the exact pathomechanism remains unclear. Furthermore, the authors found stronger associations in the cold season than in warmer months. Those findings were consistent with (43) that indicated the synergistic effect of cold spells (temperature below the 6th centile for at least three days) and high AQI (air quality index; higher mean worse quality). That study also supported the delayed type of response hypothesis – the effect on the 2nd day was higher than that on the 1st day. Correspondingly, the effect on the 3rd and subsequent days of a cold spell was higher than that on the 2nd day (43). Analogous results on the negative impact of cold spells on anxiety symptoms are reviewed above (31).

Analyses of exposure-response curves for PM10, SO2, and NO2 at lag0 with outpatient schizophrenia visits by Liang et al. found the strongest associations for PM10 concentration higher than 200 μg/m3 (higher than 700 μg/m3 did not correlate with increased number of visits), for SO2 higher than 50 μg/m3 (no upper threshold was found), and for NO2 higher than 60 μg/m3 (higher than 120 μg/m3 did not correlate with increased number of visits) (44). Authors have also shown that NO2 and SO2 were more strongly associated with outpatient visits due to schizophrenia than PM10 (44). In another study, Bai et al. proposed a prominently lower threshold for NO2 concentration at 21.5 μg/m3 (45). Thus, we conclude that the detrimental effect of air pollutants on schizophrenia patients is dose-dependent, and the established alert threshold should be as low as possible.

The relationship between the gut microbiome and schizophrenia has recently been subject to substantial attention. Dysbiosis may drive inflammatory processes and contribute to the impairment of epithelial barrier function in schizophrenia (46). It was found that exposure to air pollutants, especially long-term exposure to NO2, can cause liver dysfunction. Yi et al. found that the abundance of *Coriobacteriales* significantly correlated with increased gamma-glutamyl transpeptidase (GGT) and glutamic pyruvic transaminase (GPT) levels in patients with schizophrenia (47). Results of a study based on whole-exome sequencing show that O3 exposure is associated with lower gut microbial diversity and higher *Bacteroides caecimuris* (48). Nevertheless, to date, conducted clinical trials on psychosis microbiome research comprised methodology limitations (49, 50). Future studies should address the critical questions presented by Kelly et al. (50).

Epigenetic dysregulation is one of the possible mechanisms linking air pollution to schizophrenia risk. Numerous postmortem studies have presented alterations in DNA methylation of schizophrenia patients (51–55). Methylome-wide analysis of DNA methylation has shown 112 hypermethylated and 125 hypomethylated regions in patients with schizophrenia (56). In sum, the hypomethylated group displayed enrichment associated with immune response signaling via Notch/HH/Wnt, whereas the hypermethylated group showed enrichment connected with GPCR signaling through MAPK (56) (**Table 2**).

**Table 2:** Overview of studies on air pollution and schizophrenia among various populations.

Study group	Year of publication	Country	N° of patients	End-point/scale assessed	Proposed mechanism/hypothesis	Time of exposure	Influence on disease incidence	Ref.
general population	2022	China	10,893	N° of hospital admissions	↑acute systemic inflammation ↑oxidative stress ↑lipid peroxidation	short-term	10 μg/m3 increases in PM2.5 (RR 1.0160, 95% CI 1.0038–1.0282); PM10 (RR 1.0097, 95% CI 1.0018–1.0177), SO2 (RR 1.0738, 95% CI 1.0222–1.1280), and CO (RR 1.0013, 95% CI 1.0001–1.0026) at lag5 were statistically significant; especially SO2 was strongly associated with increased risk of schizophrenia admissions	(39)
general population	2020	China	14,650	N° of hospital admissions	↑inflammation ↑oxidative stress ↑microglial activation ↑dopamine neurotoxicity	short-term-	PM2.5 wave was defined as ≥3 consecutive days with a concentration of PM2.5 ≥90th, ≥92.5th, ≥95th, and ≥97.5th percentile; corresponded to 5.0% (2.3%-7.8%), 5.1% (1.9%-8.4%), 6.9% (3.0%-10.8%) and 12.0% (5.3%-19.1%) risk of schizophrenia hospitalization, respectively; the most significant associations were observed on the sixth day	(40)
general population	2019	China	34,865	N° of outpatient visits	↑inflammation ↑oxidative stress ↑endothelial dysfunction ↑neuronal apoptosis ↑mitochondrial dysfunction ↑crosslinks of DNA-protein	short-term	10 µg/m3 increase of PM10, SO2, and NO2 concentrations corresponded to 0.289% (95%Cl: 57 0.118%, 0.460%), 1.374% (95%Cl: 0.723%, 2.025%), and 1.881% (95%Cl: 0.957%, 58 2.805%) elevation in outpatient-visits for schizophrenia at lag 0	(44)
patients with schizophrenia	2021	China	248	liver function (GGT and GPT) depending on the gut microbiome	↑inflammation ↑blood-brain barrier dysfunction	long-term	*Coriobacteriales* mediated 13.98% and 49.56% of the associations of long-term exposure to NO2 with GGT and GPT elevation	(47)
patients with schizophrenia	2021	China	6220	N° of hospital re-admissions	↑inflammation ↑oxidative stress ↑neuronal toxicity	short-term	the increase in PM concentrations was correlated with an increased risk of schizophrenia re-admission (RRs were 1.07 (95% CI: 1.02–1.11) for PM10 and 1.05 (95% CI: 1.01–1.09) for PM2.5 at lag3	(41)
patients with schizophrenia aged ≥65 years old	2022	United States	165,572	N° of hospital admissions	↑inflammation ↑glucocorticoid activity and stress hormone cortisol concentrations ↑neuronal atrophy	short-term	in the cold season (January to March plus October to December), each increase of 5 μg/m³ increase in PM2.5 was associated with an increase in hospital admission rates of 0.77% (0.11–1.44); each increase of 5 ppb in NO2 was associated with an increase of 0.64% (0.03-0.66) increase in hospital admission rates	(42)
patients with schizophrenia aged ≥20 years old	2018	Japan	1,193	BPRS (Brief Psychiatric Rating Scale)	↑inflammation ↑oxidative stress ↑microglial activation	short-term	a significant association between BPRS ≥50 and PM2.5 concentration for individuals older than 65 years old	(57)
	2019	China	11,373	N° of hospital admissions	↑inflammation ↑oxidative stress ↑microglial activation ↓neurogenesis	short-term	the estimated RR per increase in NO2 by IQR in lag 01 was 1.10 (95% CI 1.01 to 1.18); a greater association was observed in young patients (RR 1.11, 95% CI 1.02 to 1.19).	(45)

CI, confidence interval; RR, relative risk, GGT, gamma-glutamyl transpeptidase; GPT, glutamic pyruvic transaminase; IQR, inter-quartile range; ppb, parts per billion; BPRS, Brief Psychiatric Rating Scale.

### 3.3 Air pollution and autism spectrum disorders

Numerous studies and meta-analyses indicate a relationship between exposure to air pollution and the development of ASDs. These studies focus on assessing exposure to hazardous factors in relation to exposure windows, including prenatal periods (first trimester, third trimester, and entire pregnancy), the first year after birth, and the second year after birth (58). There is no consensus as to which exposure period is most relevant - some authors suggest the period of pregnancy, others early postnatal periods (58, 59).

**Figure 2:**
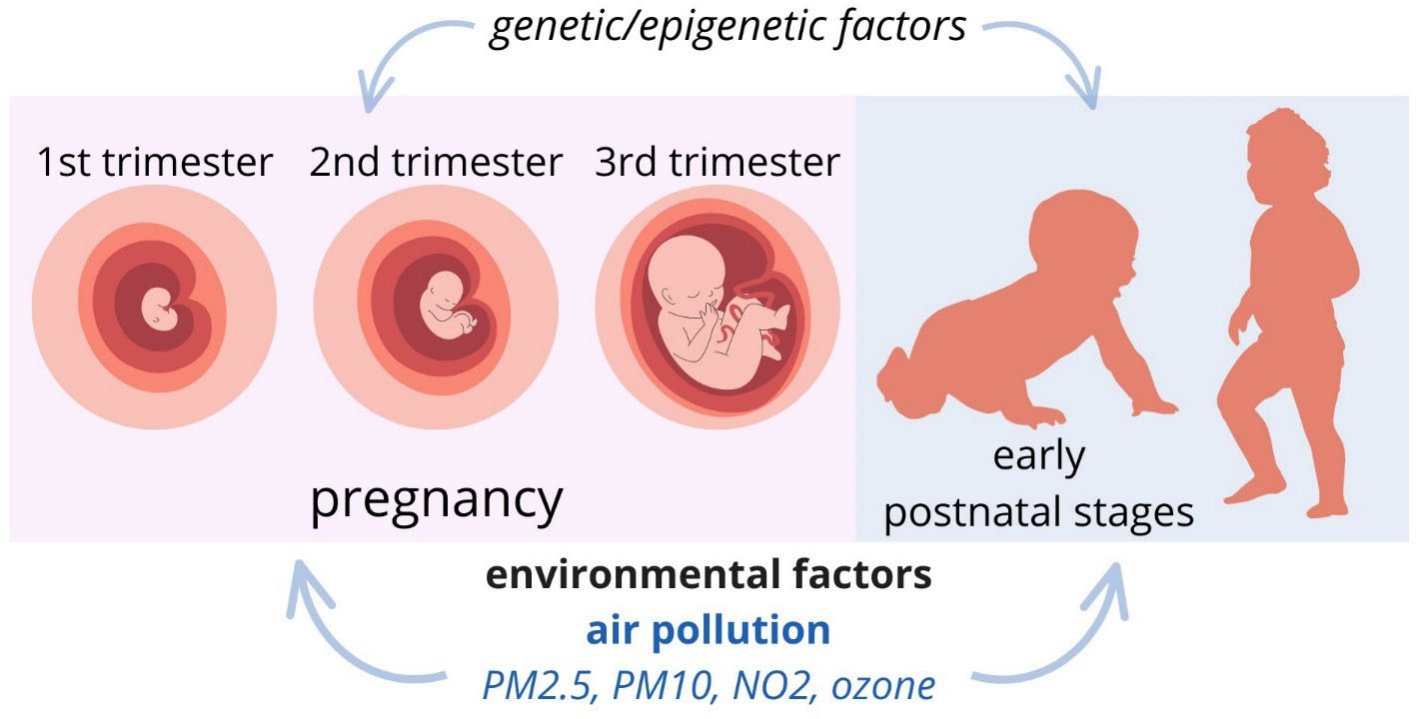
Putative exposure windows for the development of ASDs: pregnancy and early postnatal stages.

Research indicates that exposure during pregnancy to pollutants, notably PM2.5, negatively impacts the development of ASDs in newborns (60, 61). Chun et al., in meta-analysis show weak evidence for NO2 and little evidence for PM10 and ozone (62). A cohort study from Sweden shows that small-scale residential heating (mainly wood burning) and road traffic (tailpipe emissions and vehicle wear-and-tear) are relevant exposure sources (63). Yu et al. hypothesized that synergistic associations of prenatal air pollution and conditions related to maternal immune activation would increase ASD risk in children. However, there were no statistically significant interactions between MIA conditions and prenatal PM2.5 exposure on ASD risk (64). Another cohort study from Canada showed that prenatal greenspace exposure was associated with reduced odds of ASD, but in the additive scale, this effect was null at the population level (65) (**Figure 2**).

### 3.4 Air pollution and depression

Air pollution is a cause of neuroinflammation and oxidative stress that lead to cerebrovascular damage, as well as neurotransmitter and hormonal dysregulation. Due to the negative effect on the human organism, air pollution increases the risk of depression (24). Furthermore, the result of the study conducted by Fu et al. shows that air pollution has an additive effect on the development of depression in participants with a high genetic risk of that disorder (66).

Despite an enormous number of studies aimed at determining which air pollutant plays the most important role in the onset of depression, the consensus on the matter of scientists is not clear (24, 67, 68). The meta-analysis conducted by Fan et al. evaluated the association between air pollution and depression based on 22 studies from 10 different countries. The influence of ambient particulate (PM10, PM2.5) and gaseous (NO, NO2, CO, SO2, O3) air pollutants on the disorder was analyzed. The association between short-term exposure to NO2 and augmented risk of depression was observed. However, long-term and short-term exposure to other investigated air pollutants was not significantly connected with the increased risk of that disorder (67). The meta-analysis conducted by Borroni et al. examined 39 studies from different countries. The relationship between short-term and long-term exposure to PM10, PM2.5, NO2, SO2, O3, CO, and depression was evaluated. The results of the meta-analysis showed a notable association between that disorder and long-term exposure to PM2.5 and NO2. Per each 10 μg/m3 increase in PM2.5, the risk of depression was about 7% higher. In reference to long-term exposure to NO2, the risk of depression was estimated to be 4% greater per each 10 μg/m3 increase in pollutant concentration. The augmented risk of depression was also observed in short-term exposure to PM10, PM2.5, NO2, SO2, O3, and CO. However, the quality of evidence for each air pollutant (in reference to short-term exposure) was not higher than moderate (24).

Zijlema et al. investigated the association between air pollution and depressed mood in 70 928 individuals by analyzing data obtained from LifeLines (the Netherlands), KORA (Germany), HUNT (Norway) and FINRISK (Finland). Depressed mood ranged from 1.6% (KORA) to 11.3% (FINRISK). The results of the study were heterogeneous and the notable relation between depression and exposure to AAP were not found (69). The meta-analysis conducted by Zeng et al. showed that long-term exposure to PM2.5 and short-term exposure to PM10, NO2, SO2, CO is significantly related to augmented risk of depression (70).

A prospective population-based cohort study based on data from the UK Biobank cohort evaluated participants at baseline and during a follow-up. The elevated levels of five air pollutants (PM2.5, PM10, NO2, NOx) were associated with a greater risk of developing depression at baseline. However, during the follow-up, the higher odds of mental disorders were not observed for PMcoarse (2.5-10 μg). Besides, the other four evaluated air pollutants were related to increased risk of mental disorders. The results showed that long-term exposure to NO2 and NOx was more strongly associated with depression than PM2.5 (68). In another study based on the UK Biobank, incidents of depression and anxiety associated with long-term exposure to PM2.5, PM10, NO, and NO2 were evaluated. Accordingly, the exposure-response curves were non-linear. The slope was steeper at lower concentrations, with plateauing trends at higher concentrations (71).

The research conducted by Latham et al. evaluated the correlation between childhood exposure to NO2, NOx, PM2.5, PM10 and depression in UK adolescents. They examined 2232 individuals at age 18 that were exposed to high levels of ambient air pollution at age 10. The findings of the research showed that the augmented risk of developing major depressive disorder applies especially to the participants with the highest level of annual exposure to NOx and PM2.5. However, future research should include other socio-economic risk factors and genetic susceptibility alongside. This would allow improving the performance of the risk prediction model (72).

The findings of Qiu et al. study suggest that long-term exposure to PM2.5, NO2, and O3 contributes to the onset of late-life depression. The mean age of examined individuals at entry (after 5-years washout period) was 73.7 years. In reference to long-term exposure, each 5-unit increase PM2.5, NO2, and O3 was associated with an adjusted percentage increase in depression risk of 0.91% (95% CI, 0.02%-1.81%), 0.61% (95% CI, 0.31%- 0.92%), and 2.13% (95% CI, 1.63%-2.64%). Furthermore, older adults with comorbidities had a higher risk of developing late-life depression when exposed to air pollutants. Individuals with cardiovascular, metabolic, respiratory, and neurological diseases were particularly more sensitive to NO2 (73).

Additionally, it is suggested that women with impaired cognition are at increased risk of depression when exposed to air pollution (74). Lim et al. also investigated the association between exposure to air pollution and late-life depression. They used the Korean version of the Geriatric Depression Scale-Short Form (SGDS-K) to examine depressive symptomatology during a 3-year follow-up study. According to that research the exposure to PM10, NO2, O3 is related to increased risk of developing late-life depression (75).

Additionally, exposure to PM2.5 during pregnancy was associated with an augmented risk of prenatal anxiety or depression. Pregnant women are more vulnerable to gains in the concentration of air pollutants since their ventilation rate increases (76).

## 4 Light pollution

**Figure 3:**
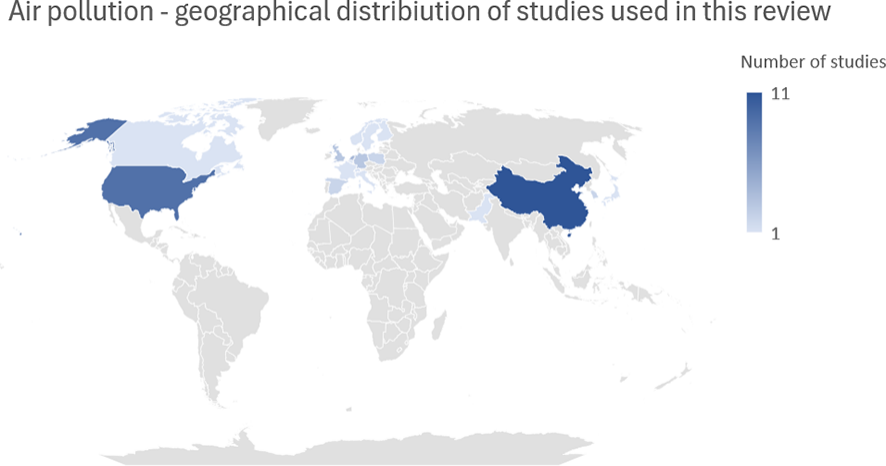
Geographical distribution of literature concerning air pollution.

According to the International Dark Sky Association, light pollution involves the improper or excessive use of artificial lighting, leading to significant environmental consequences for humans, wildlife, and climate. A staggering 80% of the global population is affected by excessive levels of artificial light. Research links light pollution to health issues, including multiple mental disorders (77–79). Outdoor and indoor dim artificial light at night (dLAN), usually around 5-10 lux, plays a key role in light pollution’s impact on neurodegeneration (80, 81), often exceeding the minimum light pollution threshold in many countries (82) (**Figure 3**).

### 4.1 Light pollution and Alzheimer’s disease

Light pollution may contribute to neurodegenerative diseases like Alzheimer’s disease (AD) or other types of dementia due to its impact on sleep, essential for neuron health. Previous research showed that there is a linear or unprecise association with late-onset dementia and dLAN (83). Artificial lighting interferes with the functioning of our internal biological clock (84, 85). Consequently, one of the most significant environmental challenges is light pollution, which has the potential to cause persistent disturbances in the circadian rhythms (81, 86). There is evidence to indicate that the disruption of circadian rhythms and associated sleep deprivation may play a pivotal role in the onset of AD (87–89). People experiencing such disruptions have a 1.49 times greater risk of developing AD compared to individuals without sleep and circadian disturbances (90).

An altered pattern of melatonin release may have a significant impact on the development of AD. This disruption might hinder the processing of amyloid precursor protein (APP) and the production of Aβ through glymphatic-lymphatic pathways and degradation mechanisms. In circumstances characterized by circadian rhythm disruption, there is a propensity for the accumulation of neurotoxic proteins within the cerebral tissue due to impaired clearance mechanisms involving glial phagocytosis and active transport. These processes are further exacerbated by heightened orexin levels and perturbations in melatonin secretion patterns. Furthermore, compromised melatonin release supports an elevated production of amyloid-beta (Aβ) (89, 91–95).

### 4.2 Light pollution impacts affective and autism spectrum disorders

Some research has suggested that disruptions in the sleep-wake cycle caused by exposure to artificial light pollution may serve as triggering factors in bipolar disorder (96–98). Furthermore, it is well established that sleep deprivation can induce manic episodes, although the precise underlying mechanisms remain unclear (99). Additionally, reactive oxygen species releasing patterns with their physiological role in seasonal photoperiodism may change under light pollution conditions. This phenomenon could contribute to the pathophysiology of bipolar disorder (100).

Numerous individuals with ASDs experience disruptions in their sleep/wake cycles, making them potentially more susceptible to the effects of circadian disruptors. dLAN alone was adequate to disturb locomotor activity patterns, intensify excessive grooming behavior, and reduce social preferences in the ASDs mouse model (101).

Several studies suggested that teenagers who had major depressive disorder displayed more severe insomnia, shorter sleep duration, greater social jetlag, reduced relative activity amplitude, and increased exposure to dLAN when compared to other groups (102, 103). Research involving animal models has demonstrated that light exposure influences behaviors resembling depression and anxiety, shedding light on the underlying neural mechanisms for these effects (104). Additionally, a separate study conducted among a representative sample of South Korean adults revealed that individuals residing in areas with higher levels of outdoor dLAN were more likely to report symptoms of depression and present suicidal behavior (105).

Exposure to dLAN during adolescence has been proposed as a factor that may lead to a slight rise in vulnerability to anxiety-related behavior in female mice and trigger depressive-like symptoms in both male and female mice (106). It was reported in the literature that anxiety disorders, especially driven by phobias, may occur in the presence of higher outdoor dLAN. This association might be explained by the fact that light pollution could lead to sleep deprivation, which is said to be directly associated with anxiety disorders (102, 107).

Remarkably, artificial light may be considered as a potential approach to certain mental disorders treatment. Many eco-friendly light bulbs, like LEDs, emit white light that contains a prominent blue component (400-490 nm), potentially disrupting circadian rhythms. Healthier options may include blue-free white light-emitting diodes (WLEDs) to counteract chronodisruption (86). Furthermore, light therapy has widely been known as a therapy for mood disorders (102, 108, 109). Crucially, altering the dLAN spectrum to longer wavelengths helped alleviate the adverse effects on activity rhythms and behaviors resembling ‘autistic’ traits in autistic mouse models (110). The mutant mice, when administered melatonin daily, exhibited a reduction in excessive grooming behavior, bringing it down to levels seen in wild-type mice, and their activity rhythms showed improvement (101). Melatonin levels reduced by exposure to light pollution could be restored by its supplementation, which may ensure neuroprotective and anxiolytic effects in the context of anxiety disorders (111). Nonetheless, as these practices are not yet widely adopted, people continue to face the issue of light pollution in their surroundings. However, alterations in daily routines and exposure to indoor lighting remain crucial factors to consider.

## 5 Noise pollution

Noise may be a perilous factor that negatively influences overall well-being, as it may affect not only the sense of hearing but also mental health. It was proven that noise induces the pituitary-adrenal-cortical axis and the sympathetic-adrenal-medullary axis. These axes may be a cause of neuroinflammation and alterations in the levels of neurotransmitters. It may also lead to the damage of synaptic plasticity (112). It was recently discovered that chronic noise exposure might lead to neurodegenerative changes in the brain due to the overproduction of molecules such as ROS. It was shown that neurons are susceptible to the harmful impact of ROS due to the increased amount of polyunsaturated fatty acids building the membranes. Neurons also have high oxygen uptake, although poor antioxidant defense (113).

There were two pathways in the noise reaction model presented. The first pathway, known as ‘‘direct’’, is characterized by the instantaneous interaction of the acoustic nerve with the various structures of the CNS. The second, called “indirect”, is related to the emotional response to the noise, which is perceived as an annoyance (114). Both pathways lead to the physiological stress reaction.

In the Dutch study, a correlation between road traffic noise and the prescription of anxiolytics was found. The study also proved the connection between psychological distress and rail traffic noise (115). Anxiety may be a result of prolonged exposure to disturbing sounds.

A positive correlation between noise and depression was found (116). The influence of noise on the risk of suicidal death was investigated in Korean patients. The research showed that prolonged exposure to noise may be a risk factor for suicide. The same study revealed that this risk is higher among adults with mental illness (117). Studies among people living in areas with high aircraft noise showed that they were more susceptible to mental disorders compared to those living in quieter places (118).

**Figure 4:**
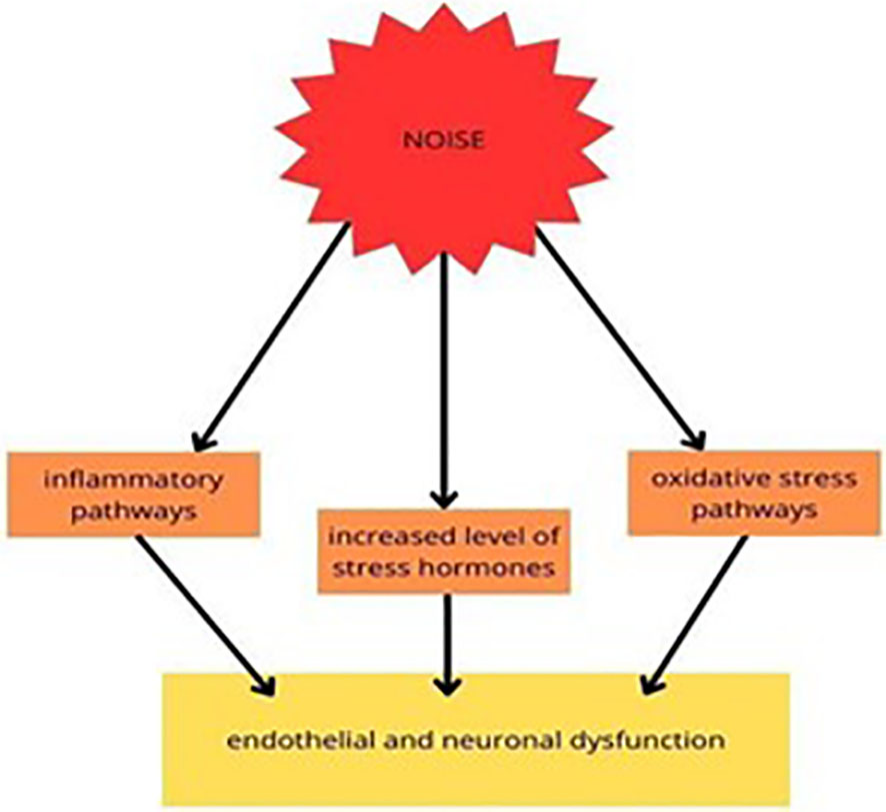
Experimental studies on the molecular pathways of traffic noise exposure proposed that it increased the level of stress hormones and mediated the inflammatory and oxidative stress (OS) pathways, resulting in endothelial and neuronal dysfunction (113).

Mental stress may also occur due to cardiovascular diseases caused by low-level noise exposure or sleep disturbance, which appears because of undesirable sounds (119). To sum up, noise has a negative influence on health and long-term exposure results in consequences both in physical and mental conditions (**Figure 4**).

## 6 Soil pollution

Along with the industrial development, soil in many places on Earth has become contaminated by many toxic substances such as crude oil, heavy metals, natural gas, agrochemicals (pesticides, herbicides, and fertilizers), polycyclic or polynuclear aromatic hydrocarbons (PAH), solvents, lead (Pb), and asbestos (120–123). These substances affect both physical and mental health.

Exposure to the Pb in the ground may lead to psychosis in humans. In one study, it was proven that the connection between the occurrence of psychosis with gene expression influenced the risk of this condition while they were exposed to Pb. It was shown that children exposed to this agent in early life may experience hyperactivation of the dopaminergic system (124). Exposure to heavy metals was also linked to the occurrence of psychosis (125). The Spanish study revealed a positive correlation between living in areas with contaminated soil by various metals and the prevalence of mental disorders. It was also proven that the group consuming vegetables more than once daily had a stronger correlation (126). Similar results were found in the Belgian population (127).

Polycyclic or polynuclear aromatic hydrocarbons (PAH) are said to be responsible for impaired neurodevelopment and behavioral changes, such as diminished ADHD (128).

Asbestos poisoning leads to the development of many mental disorders, such as depression, anxiety, and psychoticism. It was also proven that it may lead to somatization, paranoid ideation, or even hostility (129).

Considering pesticides, much research conducted in Thailand, Canada, and Brazil proved that these agents may lead to mental disorders such as depression, anxiety, and ASDs (130–133). The possible link may be the decreased activity of red blood cell cholinesterase (134).

Industrial solvents are believed to be responsible for alterations in the brain. It was proven that chronic exposure to these substances causes leukoencephalopathy and neuron degeneration. Moreover, cerebral and cerebellar atrophy caused by myelin loss, increased in size perivascular spaces and mild gliosis are observed (135).

Considering all the research described above it is certain that contaminated soil by many different substances has a negative influence on human mental life.

## 7 Water pollution

Water pollution is a problem that affects many countries, especially with higher poverty rates. There are several studies on the effects of water pollution on the environment and human health. The literature on the subject focusing on mental health studied the following pollutants: lead (Pb), arsenic (As), mercury (Hg), cadmium (Cd), chemical oxygen demand (COD), ammonia nitrogen (NH3-N), tetrachloroethylene (PCE) and volatile phenol (Fn), which were all significantly associated with mental health (136).

According to the literature, water pollution contributes to mental health deterioration and has a more significant effect on low-income subjects compared to high-income. Accordingly, water quality improvement leads to better mental health (136). Research results show that heavy metals have a greater effect on mental health compared to other agents, possibly due to their accumulation in the human body, which causes long-term effects.

One of the hypotheses explaining the correlation between pollution and mental health deterioration suggests it might be linked to inflammatory processes in response to chemicals and heavy metals (137). It is believed that high levels of heavy metals cause oxidative stress, which leads to neurotoxicity and cell apoptosis (138). Structural changes were observed in the brains of patients exposed to Pb and Cd, including decreased total cortical volume, white matter, and abnormal laminar organization.

Lead has pharmacological and morphological effects on the human nervous system. Lead exposure causes a reduction in intellectual functioning and memory reduction (138). Lead pollution is linked to an increased risk of schizophrenia and psychosis. Exposure during pregnancy and postnatal exposure is also linked to an elevated risk of ADHD and decreased learning ability (138–141).

Water is the primary source of inorganic arsenic for humans. The highest allowable limit of arsenic in drinking water set by WHO is 10μg As/liter. Exceeding this limit might be a cause of neuropsychiatric disorders, including anxiety and depression (142–144). A study conducted in Hungary associated arsenic in drinking water with a higher suicide rate (145). Arsenic can cause speech impairment and have a negative effect on cognitive and neurobehavioral performance (138).

Cadmium is an element that may be associated with increased risk for ASDs and ADHD. In the most recent study, the risk for ASD and ADHD was 1.6 times higher for children with the highest exposure compared to children with the lowest exposure (146). Nevertheless, there is a need for more data as other literature shows different results (147, 148). Cadmium is also linked to neurotoxicity and neural cell apoptosis (138).

A study evaluating the impact of prenatal and early childhood tetrachloroethylene (PCE) exposure revealed a 1.8-fold increased risk of bipolar disorder (95% CI: 0.9-3.5), 1.5-fold increased risk of PTSD (95% CI: 0.9-2.5), and 2.1-fold increased risk of schizophrenia (95% CI: 0.2-20.0). However, as the study group with schizophrenia was small (n=3), further studies are needed to corroborate these findings (149).

With the increased use of pharmaceuticals, studies have found that they are increasingly detected in surface, ground, and drinking water. Most pharmaceuticals as single compounds do not pose a threat or can be a moderate environmental threat. So far, pharmaceutical water pollution is not associated with health risks for humans due to the low doses found in tested water (150). Nevertheless, it remains a topic to be further studied. Some authors report an increase in psychoactive drug concentrations in the water supply, which might influence brain development as they can cross intestinal and placental barriers. That, in turn, may contribute to the development of neurological disorders like ASDs and AD (151).

**Figure 5:**
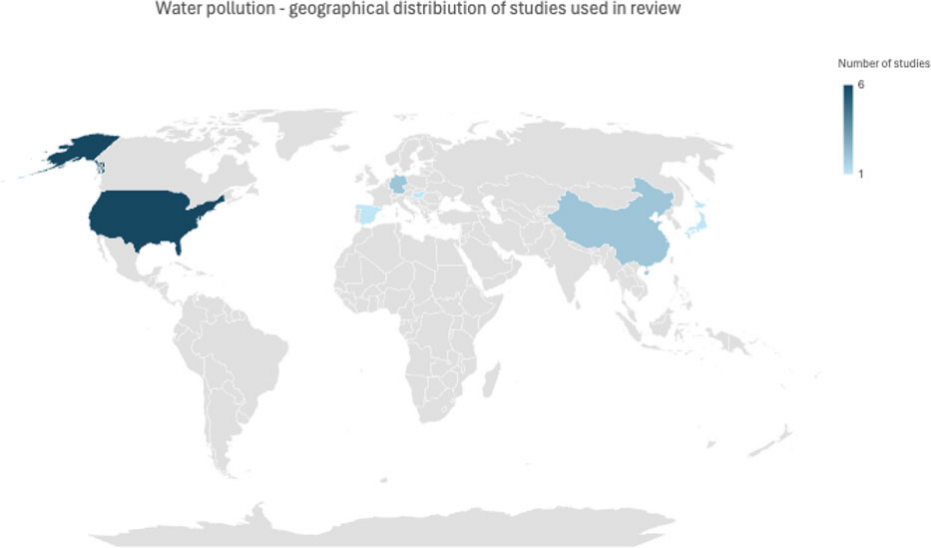
Geographical distribution of literature concerning water pollution.

Worth acknowledging is also the effect of events like oil spills, which cause immense damage to the environment and can influence mental health. A study from 2011 on the effects of the DWH (Deepwater Horizon) oil spill investigated a population already sensitized after Hurricane Katrina in 2005 when the oil spill happened, which accentuated the effects of the oil spill in 2010. Symptoms that affected the interviewed population included anger, anxiety, symptoms of GAD, and acute stress reactions with early symptoms of PTSD. A significant increase in anxiety, PTSD, and depression was also found in a study after the Exxon Valdez oil spill in 1989 (152). In both studies, a relationship between the oil spill and increased alcohol and substance use was found. Studies done years after the oil spill suggest that the effects on mental health persist after many years (152, 153). Similar outcomes were observed in Flint, Michigan, where issues with water contamination caused an increase in stress, anxiety, and depression (154). Some studies indicate a less significant effect on mental health, especially in groups with good social support and satisfaction with recovery aid (155) (**Figure 5**).

## 8 Extreme weather conditions

As the prevalence of extreme weather events increases with climate change, we cannot ignore the effect it has on mental health. Extreme weather events include floods, hurricanes, wildfires, and droughts. According to the literature, the most common mental health effects are depression, PTSD, and anxiety (156). These disorders are especially concerning for the most vulnerable groups with risk factors, including females, children [up to 71% of children experience PTSD symptoms as a result of natural disasters (157)], minorities, people with low socioeconomic status and education or with preexisting mental health symptomatology and high exposure to extreme weather conditions (156, 158, 159). Studies suggest that repeated exposure to extreme weather events like hurricanes can have cumulative effects on mental health, causing deterioration of mental health symptoms (160). These effects may also occur with indirect exposure through knowing someone who experienced stress due to extreme weather events or being exposed to them through media (156, 161). Authors have also suggested that natural disasters influence alcohol use, but the outcomes are so far unclear (156).

Research shows an association between a higher level of exposure to hurricanes with a higher risk of PD (panic disorder), PTSD, depression, or anxiety (162) and a cumulative effect of repeated hurricane exposure, including indirect exposure through media, which can lead to sensitization and more adverse distress symptoms (160). Factors like pre-existing mental illness, property loss, and injury before or during a hurricane were positively associated with short-term psychological distress (160, 163). A different outcome was observed in a study from Puerto Rico. In this study, the data was not statistically significant and did not entail mental health deterioration with property loss (162). In longitudinal studies, research suggests the effects of hurricane-related trauma persist and can affect 16,7% of responders (164). Populations exposed to hurricanes had the highest rate of PTSD symptoms compared to other extreme weather exposures (165).

Floods are one of the most common natural disasters occurring in the world. A series of recent studies show an elevated risk of depression, anxiety, and PTSD among groups exposed to flood (166). In the long-term, subjects experienced anxiety (>60%), increased stress level (<40%), frequent flashbacks (23%), sleeplessness (18%), depression (18%), and nightmares (<10%) (167). Meta-analysis reports that the combined incidence of PTSD among flood victims reaches 15.74% and can vary significantly across studies (0.63%-46.64%). The reasons for this disproportion are suggested to be cultural background, different sample sizes, diagnostic criteria, period, and post-flood interventions (166) 78.6% of people exposed to flood in Tamil Nadu reported no access to food, and 37.7% had no access to drinking water which resulted in higher levels of anxiety and depression. Rates were also higher in the group who experienced material or personal loss. Different levels of depression were observed among 45.29% of the study participants. The severity of depression correlated with a higher level of exposure and intensity of flood (166, 168).

Research shows that wildfires contribute to mental distress, fear, and feelings of uncertainty, which can cause long-term mental problems (169). Extended exposure to wildfire and smoke may cause an increased risk of ever having a depressive episode and anxiety (170). Exposure to wildfires can also result in PTSD symptoms. In particular, direct exposure was found to be positively correlated with PTSD and depression symptoms (171). For this reason, firefighters and patients with burns are significantly affected by mental health issues after the exposition (172). Factors contributing to resilience, including sleep quality, physical exercise, mindfulness, and emotional support, were found to negatively correlate with PTSD, depression, and anxiety symptom severity (171).

**Figure 6:**
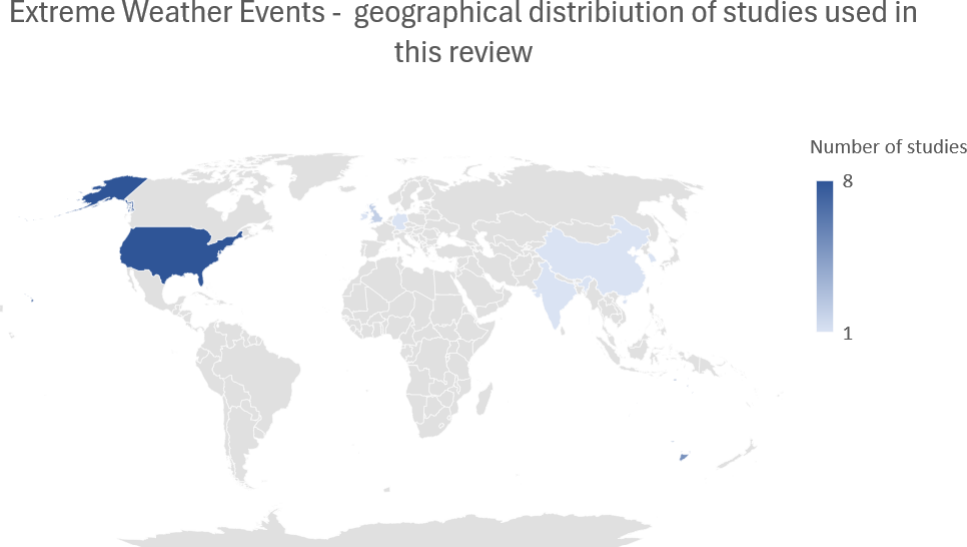
Geographical distribution of literature concerning extreme weather conditions.

Droughts have a more gradual effect than other extreme weather events. They have been linked to increased suicide risk among male farmers (173) and increased general mental distress, especially in young and middle-aged women (174). In a study by Hanigan et al., older women had lower levels of distress compared to young and middle-aged women, which may be linked to experience and growing up in low-income communities. They were also more involved in volunteer work and social system communities (174). Contrary to previous studies that suggested a positive association between distress and farming occupation. Hanigan et al. did not find different levels of distress between farmers and non-farmers participants. Most studies come from rural areas of Australia. Different outcomes were observed in urban areas where the impact of drought on mental health was significantly lower (175, 176) (**Figure 6**; **Table 3**).

**Table 3:** Overview of studies on extreme weather exposure and its mental health consequences.

Ref.	Study location	Number of subjects	Extreme weather exposure	Mental health effect
(160)	Florida, USA	2873	Hurricane	increased risk of PTSS, distress, the cumulative effect of repeated exposure
(162)	Puerto Rico	456	Hurricane	hurricane exposure is associated with an increased risk of depression symptoms (10%), PTSD (8%), and GAD (10%)
(164)	New Orleans, USA	942	Hurricane	PTTS declined after every follow-up, but the risk of PTSS remains higher after 12 years compared to the group without hurricane-related trauma; PD remained consistently higher compared to the pre-disaster period
(163)	New York, Long Island, USA	130	Hurricane	increased risk of PTSD symptoms after 1-year follow-up; symptoms of depression and anxiety decreased at follow-up
(172)	Australia	2883	Bushfire	among the Australian population affected by bushfires, firefighters were the group with the highest prevalence of longitudinal mental health issues; mental health problems decreased with time
(170)	Oregon, USA	5807	Wildfire	increased feeling of worry in adults, increased risk of ever having a depressive episode
(177)	Gangwon Province, South Korea	206	Wildfire	insomnia (59,2%), anxiety (50%), chest tightness (34%), grief (33%), flashbacks (33%), and depression (32,5%) among wildfire victims.
(171)	California USA	725	Wildfire	direct exposure to large-scale fires significantly increases the risk of mental health disorders, particularly PTSD and depression
(178)	England	7525	Storm, flood	positive association found for CMD, suicidal ideation, and the number of suicide attempts
(168)	Tamil Nadu, India	223	Flood	26,9% of the subjects affected by the flood showed symptoms of PTSD; 27,4% reported symptoms consistent with anxiety disorder; depression was observed among 45,29% of subjects
(166)	Various locations	40 600	Flood	15.74% incidence of PTSD in flood victims; type, severity, and duration of exposure are the most important risk factors for PTSD
(179)	New South Wales, Australia	2607	Drought	decrees of drought-related distress over time (2,5-3 years)
(174)	Victoria, Australia	5312	Drought	a statistically significant association between distress and drought duration in young and middle-aged rural women regardless of farming occupation
(180)	Rural and regional areas of Australia	8000	Drought	higher rate of mental health problems in areas in drought (13.3%) than in areas not in drought (10.8%), and the mental health well-being score is significantly lower in areas in drought (72.7) than in areas not in drought (75.5).
(175)	Australia	5012	Drought	long periods of drought (20-32 months) were associated with increased distress in rural areas; similar effects of drought were not observed in urban areas

PTSS, posttraumatic stress symptoms; PTSD, posttraumatic stress disorder; GAD, general anxiety disorder; CMD, common mental disorder; PD, panic disorder.

## 9 Discussion

Environmental pollution remains a matter of great significance to public health in the modern world. Several environmental exposures, including air, soil, and water pollution, have been associated with adverse physical and mental health outcomes, as well as premature deaths. Light and noise pollution are underestimated concerns for citizens in urbanized areas. Extreme weather conditions, such as hurricanes, floods, wildfires, and droughts, also may implicate serious long-term issues.

Exposure-response curves between air pollutants and mental disorders prevalence were non-linear. The slope was steeper at lower concentrations, with plateauing trends at higher concentrations. Increased concentrations of PM2.5, NO2, and SO2 were the most detrimental, particularly for vulnerable groups, namely children, pregnant women, and elderly patients with comorbidities. Increased concentrations of these air pollutants were the most strongly associated with exacerbation of anxiety, schizophrenia, and depression symptoms, as well as increased number of hospital and outpatient disease-specific visits. For PM10 studies were not consistent, PM10 could be a confounding factor that worsens symptoms with co-existence of other air pollutants. Several lines of evidence suggest that early-life exposure to air pollution may cause increased incidence of schizophrenia, anxiety, and depression in late-life, although studies are equivocal. A causal relationships cannot be excluded. However, more research is needed to corroborate those associations.

Research highlights air pollution as a significant factor in the development of autism, emphasizing that pollutants may pose greater risks during certain stages of pregnancy and early postnatal life. PM2.5 has been particularly singled out, but studies also point to NO2, PM10, and ozone as contributing factors, with an emphasis on sources such as residential heating and road traffic. Moreover, in the case of ASDs, studies propose a synergistic association between prenatal air pollution exposure and the activation of the maternal immune system. However, statistically significant interactions remain inconclusive.

To date, a pathomechanism linking air pollutants to mental health deterioration has not been fully elucidated, albeit there are many hypotheses. Oxidative stress, systemic inflammation, epigenetic dysregulation, and disruption of the blood-brain barrier were the most commonly suggested pathomechanisms linked to mental health disorders. These processes lead to disturbance in neurotransmitters, e.g., dopaminergic or glutaminergic toxicity, increased glucocorticoid activity, increased microglial activation, and reduced neurogenesis. Thus, we conclude that the detrimental effect of air pollutants on mental health is multifactorial.

Light pollution, stemming from improper or excessive artificial lighting, affects a significant portion of the global population, leading to various adverse health outcomes, particularly in relation to neurodegenerative diseases like AD, bipolar disorder, ASDs, depression, and anxiety disorders. Studies identify disruptions in circadian rhythms, sleep patterns, and hormone release as key mechanisms through which light pollution influences mental health. Whether it’s the disturbance of sleep-wake cycles, alterations in melatonin secretion, or the accumulation of neurotoxic proteins, the effects of light pollution on mental well-being manifest across diverse populations and conditions. Moreover, research consistently suggests potential therapeutic approaches, such as using eco-friendly lighting options and light therapy, to mitigate the adverse effects of light pollution on mental health. While challenges remain in implementing widespread mitigation strategies, the scientific consensus underscores the urgent need to address light pollution to safeguard public mental health effectively.

It was recently discovered that chronic noise exposure may lead to neurodegenerative changes in the brain due to the harmful impact of ROS. A positive correlation between noise and depression was found, as well as between noise and suicide rate.

Crude oil, heavy metals, natural gas, agrochemicals (pesticides, herbicides, and fertilizers), polycyclic or polynuclear aromatic hydrocarbons (PAH), solvents, lead (Pb), and asbestos are substances present in the soil which have a negative influence on human mental health such as psychosis, impaired neurodevelopment and behavioral changes, depression, anxiety, and ASDs.

Hurricanes, floods, wildfires, and droughts are all major stressors that can contribute to developing mental health disorders, especially depression, PTSD and anxiety. Deeper knowledge about the subject could enable early response in the most affected groups and, consequently, reduce mental health symptoms. Most vulnerable groups include females, children, minorities, people with low socioeconomic status, and pre-existing mental illness.

The most common water pollutants known for mental health deterioration are heavy metals due to their neurotoxic effect. Exposure to heavy metals may lead to a variety of symptoms, including psychosis, schizophrenia, and depression. In affected children, studies suggest an increased risk of ADHD and ASDs.

Several limitations should be acknowledged. First, the methodology of different studies was not homogenous. The authors utilized various scales and end-points for symptom severity assessment. Hence, we were unable to conduct a meta-analysis, and our review can be considered as a comprehensive narrative review. Second, in the majority of studies, data was obtained from single-country or single-region areas or comprised of information from selected populations. That implies an increased risk of selection bias. Third, in different studies, authors stated that measurement of specific pollutants was impossible, suggesting possible measurement bias. Nevertheless, we believe that these limitations are strictly related to the observational type of reviewed studies, and their findings should not be omitted.

## Author contributions

MT: Conceptualization, Methodology, Writing – original draft, Writing – review & editing. JK: Methodology, Writing – original draft, Writing – review & editing. SK: Writing – original draft, Writing – review & editing. NP: Writing – original draft, Writing – review & editing. MP: Writing – original draft, Writing – review & editing. KM: Writing – original draft, Writing – review & editing. PP: Funding acquisition, Supervision, Validation, Writing – review & editing.

## References

[1] Ventriglio A Bellomo A Di Gioia I Di Sabatino D Favale D De Berardis D. Environmental pollution and mental health: a narrative review of literature. CNS Spectr. (2021) 26:51–61. doi: 10.1017/S1092852920001303 32284087

[2] Szuhany KL Simon NM. Anxiety disorders: A review. JAMA. (2022) 328:2431–45. doi: 10.1001/JAMA.2022.22744 36573969

[3] Carmin C L Ownby R. Assessment of anxiety in older adults. Handb Assess Clin Gerontology. (2010) 2:45–60. doi: 10.1016/B978-0-12-374961-1.10002-8

[4] Wilmer MT Anderson K Reynolds M. Correlates of quality of life in anxiety disorders: review of recent research. Curr Psychiatry Rep. (2021) 23:3. doi: 10.1007/S11920-021-01290-4 34613508 PMC8493947

[5] Zhou YM Fan YN Yao CY Xu C Liu XL Li X. Association between short-term ambient air pollution and outpatient visits of anxiety: A hospital-based study in northwestern China. Environ Res. (2021) 197:111071. doi: 10.1016/J.ENVRES.2021.111071 33798515

[6] Velligan DI Rao S. The epidemiology and global burden of schizophrenia. J Clin Psychiatry. (2023) 84:MS21078COM5. doi: 10.4088/JCP.MS21078COM5 36652681

[7] Schwab SG Wildenauer DB. Genetics of psychiatric disorders in the GWAS era: an update on schizophrenia. Eur Arch Psychiatry Clin Neurosci. (2013) 263 Suppl 2:S147–54. doi: 10.1007/s00406-013-0450-z 24071914

[8] Hilker R Helenius D Fagerlund B Skytthe A Christensen K Werge TM. Heritability of schizophrenia and schizophrenia spectrum based on the nationwide danish twin register. Biol Psychiatry. (2018) 83:492–8. doi: 10.1016/J.BIOPSYCH.2017.08.017 28987712

[9] Light G Greenwood TA Swerdlow NR Calkins ME Freedman R Green MF. Comparison of the heritability of schizophrenia and endophenotypes in the COGS-1 family study. Schizophr Bull. (2014) 40:1404–11. doi: 10.1093/SCHBUL/SBU064 PMC4193725 24903414

[10] Robinson N Bergen SE. Environmental risk factors for schizophrenia and bipolar disorder and their relationship to genetic risk: current knowledge and future directions. Front Genet. (2021) 12:686666/BIBTEX. doi: 10.3389/fgene.2021.686666 34262598 PMC8273311

[11] Cheroni C Caporale N Testa G. Autism spectrum disorder at the crossroad between genes and environment: contributions, convergences, and interactions in ASD developmental pathophysiology. Mol Autism 2020 11:1. (2020) 11:1–18. doi: 10.1186/S13229-020-00370-1 PMC7488083 32912338

[12] Yin J Schaaf CP. Autism genetics - an overview. Prenat Diagn. (2017) 37:14–30. doi: 10.1002/PD.4942 27743394

[13] Donovan APA Basson MA. The neuroanatomy of autism - a developmental perspective. J Anat. (2017) 230:4–15. doi: 10.1111/JOA.12542 27620360 PMC5192959

[14] Gładka A Rymaszewska J Zatoński T. Impact of air pollution on depression and suicide. Int J Occup Med Environ Health. (2018) 31:711–21. doi: 10.13075/IJOMEH.1896.01277 30281038

[15] Gomułka K Liebhart J Mędrala W. Vascular endothelial growth factor as a putative biomarker of depression in asthmatics with reversible airway narrowing. J Clin Med. (2021) 10:5301. doi: 10.3390/JCM10225301 34830591 PMC8622768

[16] Lin P Li X Liang Z Wang T. Association between depression and mortality in persons with asthma: a population-based cohort study. Allergy Asthma Clin Immunol. (2022) 18:29. doi: 10.1186/S13223-022-00672-4 35365240 PMC8973604

[17] Khomenko S Cirach M Pereira-Barboza E Mueller N Barrera-Gómez J Rojas-Rueda D. Premature mortality due to air pollution in European cities: a health impact assessment. Lancet Planet Health. (2021) 5:e121–34. doi: 10.1016/S2542-5196(20)30272-2 33482109

[18] Silva RA West JJ Lamarque JF Shindell DT Collins WJ Dalsoren S. The effect of future ambient air pollution on human premature mortality to 2100 using output from the ACCMIP model ensemble. Atmos Chem Phys. (2016) 16:9847–62. doi: 10.5194/ACP-16-9847-2016 PMC5730074 29250104

[19] Lee BJ Kim B Lee K. Air pollution exposure and cardiovascular disease. Toxicol Res. (2014) 30:71. doi: 10.5487/TR.2014.30.2.071 25071915 PMC4112067

[20] Rajak R Chattopadhyay A. Short and long term exposure to ambient air pollution and impact on health in India: A systematic review. Int J Environ Health Res. (2020) 30:593–617. doi: 10.1080/09603123.2019.1612042 31070475

[21] Niu Z Liu F Yu H Wu S Xiang H. Association between exposure to ambient air pollution and hospital admission, incidence, and mortality of stroke: an updated systematic review and meta-analysis of more than 23 million participants. Environ Health Prev Med. (2021) 26:15. doi: 10.1186/S12199-021-00937-1 33499804 PMC7839211

[22] Arias-Pérez RD Taborda NA Gómez DM Narvaez JF Porras J Hernandez JC. Inflammatory effects of particulate matter air pollution. Environ Sci pollut Res Int. (2020) 27:42390–404. doi: 10.1007/s11356-020-10574-w 32870429

[23] Hahad O Lelieveld J Birklein F Lieb K Daiber A Münzel T. Ambient air pollution increases the risk of cerebrovascular and neuropsychiatric disorders through induction of inflammation and oxidative stress. Int J Mol Sci. (2020) 21:1–24. doi: 10.3390/IJMS21124306 PMC7352229 32560306

[24] Borroni E Pesatori AC Bollati V Buoli M Carugno M. Air pollution exposure and depression: A comprehensive updated systematic review and meta-analysis. Environ Pollut. (2022) 292:118245. doi: 10.1016/J.ENVPOL.2021.118245 34600062

[25] Hautekiet P Saenen ND Demarest S Keune H Pelgrims I van der Heyden J. Air pollution in association with mental and self-rated health and the mediating effect of physical activity. Environ Health. (2022) 21:1–13. doi: 10.1186/s12940-022-00839-x 35255905 PMC8903639

[26] Ma Y Wang W Li Z Si Y Wang J Chen L. Short-term exposure to ambient air pollution and risk of daily hospital admissions for anxiety in China: A multicity study. J Hazard Mater. (2022) 424:127535. doi: 10.1016/J.JHAZMAT.2021.127535 34879525

[27] Power MC Kioumourtzoglou MA Hart JE Okereke OI Laden F Weisskopf MG. The relation between past exposure to fine particulate air pollution and prevalent anxiety: observational cohort study. BMJ. (2015) 350:h1111. doi: 10.1136/BMJ.H1111 25810495 PMC4373600

[28] Pun VC Manjourides J Suh H. Association of ambient air pollution with depressive and anxiety symptoms in older adults: results from the NSHAP study. Environ Health Perspect. (2017) 125:342–8. doi: 10.1289/EHP494 PMC5332196 27517877

[29] Abramis DJ. Relationship of job stressors to job performance: linear or an inverted-U? Psychol Rep. (1994) 75:547–58. doi: 10.2466/PR0.1994.75.1.547 7809330

[30] Bari MW Saleem S Bashir M Ahmad B. Impact of ambient air pollution on outdoor employees’ performance: Mediating role of anxiety. Front Psychol. (2022) 13:926534. doi: 10.3389/FPSYG.2022.926534 36248467 PMC9554460

[31] Li H Li M Zhang S Qian Z(Zhang Z Zhang K. Interactive effects of cold spell and air pollution on outpatient visits for anxiety in three subtropical Chinese cities. Sci Total Environ. (2022) 817:152789. doi: 10.1016/J.SCITOTENV.2021.152789 34990686 PMC8907861

[32] Lamichhane DK Jung DY Shin YJ Lee KS Lee SY Ahn K. Association of ambient air pollution with depressive and anxiety symptoms in pregnant women: A prospective cohort study. Int J Hyg Environ Health. (2021) 237:113823. doi: 10.1016/J.IJHEH.2021.113823 34364017

[33] Yolton K Khoury JC Burkle J LeMasters G Cecil K Ryan P. Lifetime exposure to traffic-related air pollution and symptoms of depression and anxiety at age 12 years. Environ Res. (2019) 173:206. doi: 10.1016/J.ENVRES.2019.03.005 PMC7388180 30925441

[34] Vert C Sánchez-Benavides G Martínez D Gotsens X Gramunt N Cirach M. Effect of long-term exposure to air pollution on anxiety and depression in adults: A cross-sectional study. Int J Hyg Environ Health. (2017) 220:1074–80. doi: 10.1016/J.IJHEH.2017.06.009 28705430

[35] Jorcano A Lubczyńska MJ Pierotti L Altug H Ballester F Cesaroni G. Prenatal and postnatal exposure to air pollution and emotional and aggressive symptoms in children from 8 European birth cohorts. Environ Int. (2019) 131:104927. doi: 10.1016/J.ENVINT.2019.104927 31326824

[36] Madrigano J Baccarelli A Mittleman MA Sparrow D Spiro A Vokonas PS. Air pollution and DNA methylation: interaction by psychological factors in the VA Normative Aging Study. Am J Epidemiol. (2012) 176:224–32. doi: 10.1093/AJE/KWR523 PMC3491965 22798479

[37] Manisalidis I Stavropoulou E Stavropoulos A Bezirtzoglou E. Environmental and health impacts of air pollution: A review. Front Public Health. (2020) 8:14. doi: 10.3389/FPUBH.2020.00014 32154200 PMC7044178

[38] Peterson BS Bansal R Sawardekar S Nati C Elgabalawy ER Hoepner LA. Prenatal exposure to air pollution is associated with altered brain structure, function, and metabolism in childhood. J Child Psychol Psychiatry. (2022) 63:1316–31. doi: 10.1111/JCPP.13578 35165899

[39] Ji Y Liu B Song J Pan R Cheng J Wang H. Short-term effects and economic burden assessment of ambient air pollution on hospitalizations for schizophrenia. Environ Sci pollut Res Int. (2022) 29:45449–60. doi: 10.1007/s11356-022-19026-z 35149942

[40] Bai L Yang J Zhang Y Zhao D Su H. Durational effect of particulate matter air pollution wave on hospital admissions for schizophrenia. Environ Res. (2020) 187:109571. doi: 10.1016/J.ENVRES.2020.109571 32416354

[41] Ji Y Liu B Song J Pan R Cheng J Su H. Wang H. Particulate matter pollution associated with schizophrenia hospital re-admissions: a time-series study in a coastal Chinese city. Environ Sci pollut Res Int. (2021) 28:58355–63. doi: 10.1007/S11356-021-14816-3 34115296

[42] Qiu X Danesh-Yazdi M Wei Y Di Q Just A Zanobetti A. Associations of short-term exposure to air pollution and increased ambient temperature with psychiatric hospital admissions in older adults in the USA: a case–crossover study. Lancet Planet Health. (2022) 6:e331–41. doi: 10.1016/S2542-5196(22)00017-1 PMC9044858 35397221

[43] He Y Zhang X Gao J Gao H Cheng J Xu Z. The impact of cold spells on schizophrenia admissions and the synergistic effect with the air quality index. Environ Res. (2022) 212:113243. doi: 10.1016/J.ENVRES.2022.113243 35398316

[44] Liang Z Xu C Cao Y Kan HD Chen RJ Yao CY. The association between short-term ambient air pollution and daily outpatient visits for schizophrenia: A hospital-based study. Environ pollut. (2019) 244:102–8. doi: 10.1016/J.ENVPOL.2018.09.142 30326384

[45] Bai L Zhang X Zhang Y Cheng Q Duan J Gao J. Ambient concentrations of NO2 and hospital admissions for schizophrenia. Occup Environ Med. (2019) 76:125–31. doi: 10.1136/OEMED-2018-105162 30366962

[46] Safadi JM Quinton AMG Lennox BR Burnet PWJ Minichino A. Gut dysbiosis in severe mental illness and chronic fatigue: a novel trans-diagnostic construct? A systematic review and meta-analysis. Mol Psychiatry. (2021) 27:141–53. doi: 10.1038/s41380-021-01032-1 PMC8960409 33558650

[47] Yi W Ji Y Gao H Pan R Wei Q Cheng J. Does the gut microbiome partially mediate the impact of air pollutants exposure on liver function? Evidence based on schizophrenia patients. Environ Pollut. (2021) 291:118135. doi: 10.1016/J.ENVPOL.2021.118135 34534831

[48] Fouladi F Bailey MJ Patterson WB Sioda M Blakley IC Fodor AA. Air pollution exposure is associated with the gut microbiome as revealed by shotgun metagenomic sequencing. Environ Int. (2020) 138:105604. doi: 10.1016/J.ENVINT.2020.105604 32135388 PMC7181344

[49] Szeligowski T Yun AL Lennox BR Burnet PWJ. The gut microbiome and schizophrenia: the current state of the field and clinical applications. Front Psychiatry. (2020) 11:156/BIBTEX. doi: 10.3389/fpsyt.2020.00156 32226399 PMC7080964

[50] Kelly JR Minuto C Cryan JF Clarke G Dinan TG. The role of the gut microbiome in the development of schizophrenia. Schizophr Res. (2021) 234:4–23. doi: 10.1016/J.SCHRES.2020.02.010 32336581

[51] Montano C Taub MA Jaffe A Briem E Feinberg JI Trygvadottir R. Association of DNA methylation differences with schizophrenia in an epigenome-wide association study. JAMA Psychiatry. (2016) 73:506–14. doi: 10.1001/JAMAPSYCHIATRY.2016.0144 PMC6353566 27074206

[52] Jaffe AE Gao Y Deep-Soboslay A Tao R Hyde TM Weinberger DR. Mapping DNA methylation across development, genotype and schizophrenia in the human frontal cortex. Nat Neurosci. (2016) 19:40–7. doi: 10.1038/NN.4181 PMC4783176 26619358

[53] Wockner LF Noble EP Lawford BR Young RMD Morris CP Whitehall VLJ. Genome-wide DNA methylation analysis of human brain tissue from schizophrenia patients. Transl Psychiatry. (2014) 4:e339. doi: 10.1038/TP.2013.111 24399042 PMC3905221

[54] Hannon E Dempster E Viana J Burrage J Smith AR Macdonald R. An integrated genetic-epigenetic analysis of schizophrenia: evidence for co-localization of genetic associations and differential DNA methylation. Genome Biol. (2016) 17:176. doi: 10.1186/s13059-016-1041-x 27572077 PMC5004279

[55] Ross CM. Epigenetics, traffic and firewood. Schizophr Res. (2009) 109:193. doi: 10.1016/J.SCHRES.2009.01.007 19217264

[56] Shen L Lv X Huang H Li M Huai C Wu X. Genome-wide analysis of DNA methylation in 106 schizophrenia family trios in Han Chinese. EBioMedicine. (2021) 72:103609. doi: 10.1016/j.ebiom.2021.103609 34628353 PMC8511801

[57] Eguchi R Onozuka D Ikeda K Kuroda K Ieiri I Hagihara A. The relationship between fine particulate matter (PM2.5) and schizophrenia severity. Int Arch Occup Environ Health. (2018) 91:613–22. doi: 10.1007/s00420-018-1311-x 29682692

[58] Lin LZ Zhan XL Jin CY Liang JH Jing J Dong GH. The epidemiological evidence linking exposure to ambient particulate matter with neurodevelopmental disorders: A systematic review and meta-analysis. Environ Res. (2022) 209:112876. doi: 10.1016/J.ENVRES.2022.112876 35134379

[59] Imbriani G Panico A Grassi T Idolo A Serio F Bagordo F. Early-life exposure to environmental air pollution and autism spectrum disorder: A review of available evidence. Int J Environ Res Public Health. (2021) 18:1204. doi: 10.3390/IJERPH18031204 33572907 PMC7908547

[60] Dutheil F Comptour A Morlon R Mermillod M Pereira B Baker JS. Autism spectrum disorder and air pollution: A systematic review and meta-analysis. Environ pollut. (2021) 278:116856. doi: 10.1016/J.ENVPOL.2021.116856 33714060

[61] Li Y Xie T Cardoso Melo RD de Vries M Lakerveld J Zijlema W. Longitudinal effects of environmental noise and air pollution exposure on autism spectrum disorder and attention-deficit/hyperactivity disorder during adolescence and early adulthood: The TRAILS study. Environ Res. (2023) 227:115704. doi: 10.1016/J.ENVRES.2023.115704 36940817

[62] Chun HK Leung C Wen SW McDonald J Shin HH. Maternal exposure to air pollution and risk of autism in children: A systematic review and meta-analysis. Environ Pollut. (2020) 256:113307. doi: 10.1016/J.ENVPOL.2019.113307 31733973

[63] Flanagan E Malmqvist E Rittner R Gustafsson P Källén K Oudin A. Exposure to local, source-specific ambient air pollution during pregnancy and autism in children: a cohort study from southern Sweden. Sci Rep. (2023) 13:1–13. doi: 10.1038/s41598-023-30877-5 36890287 PMC9995328

[64] Yu X Mostafijur Rahman M Carter SA Lin JC Zhuang Z Chow T. Prenatal air pollution, maternal immune activation, and autism spectrum disorder. Environ Int. (2023) 179:108148. doi: 10.1016/J.ENVINT.2023.108148 37595536 PMC10792527

[65] Pagalan L Oberlander TF Hanley GE Rosella LC Bickford C Weikum W. The association between prenatal greenspace exposure and Autism spectrum disorder, and the potentially mediating role of air pollution reduction: A population-based birth cohort study. Environ Int. (2022) 167:107445. doi: 10.1016/J.ENVINT.2022.107445 35921770

[66] Fu Z Liu Q Liang J Weng Z Li W Xu J. Air pollution, genetic factors and the risk of depression. Sci Total Environ. (2022) 850:158001. doi: 10.1016/J.SCITOTENV.2022.158001 35973541

[67] Fan SJ Heinrich J Bloom MS Zhao TY Shi TX Feng WR. Ambient air pollution and depression: A systematic review with meta-analysis up to 2019. Sci Total Environ. (2020) 701:134721. doi: 10.1016/J.SCITOTENV.2019.134721 31715478

[68] Gao X Jiang M Huang N Guo X Huang T. Long-term air pollution, genetic susceptibility, and the risk of depression and anxiety: A prospective study in the UK biobank cohort. Environ Health Perspect. (2023) 131:017002–1–017002–14. doi: 10.1289/EHP10391 PMC9812022 36598457

[69] Zijlema WL Wolf K Emeny R Ladwig KH Peters A Kongsgård H. The association of air pollution and depressed mood in 70,928 individuals from four European cohorts. Int J Hyg Environ Health. (2016) 219:212–9. doi: 10.1016/J.IJHEH.2015.11.006 26682644

[70] Zeng Y Lin R Liu L Liu Y Li Y. Ambient air pollution exposure and risk of depression: A systematic review and meta-analysis of observational studies. Psychiatry Res. (2019) 276:69–78. doi: 10.1016/J.PSYCHRES.2019.04.019 31029037

[71] Yang T Wang J Huang J Kelly FJ Li G. Long-term exposure to multiple ambient air pollutants and association with incident depression and anxiety. JAMA Psychiatry. (2023) 80:305–13. doi: 10.1001/JAMAPSYCHIATRY.2022.4812 PMC10077109 36723924

[72] Latham RM Kieling C Arseneault L Botter-Maio Rocha T Beddows A Beevers SD. Childhood exposure to ambient air pollution and predicting individual risk of depression onset in UK adolescents. J Psychiatr Res. (2021) 138:60–7. doi: 10.1016/J.JPSYCHIRES.2021.03.042 PMC8412033 33831678

[73] Qiu X Shi L Kubzansky LD Wei Y Castro E Li H. Association of long-term exposure to air pollution with late-life depression in older adults in the US. JAMA Netw Open. (2023) 6:e2253668. doi: 10.1001/JAMANETWORKOPEN.2022.53668 36763364 PMC9918878

[74] Altuğ H Fuks KB Hüls A Mayer AK Tham R Krutmann J. Air pollution is associated with depressive symptoms in elderly women with cognitive impairment. Environ Int. (2020) 136:105448. doi: 10.1016/J.ENVINT.2019.105448 31931346

[75] Lim YH Kim H Kim JH Bae S Park HY Hong YC. Air pollution and symptoms of depression in elderly adults. Environ Health Perspect. (2012) 120:1023–8. doi: 10.1289/EHP.1104100 PMC3404652 22514209

[76] Zhao W Zhao Y Wang P Zhou Y Meng X Ma W. PM2.5 exposure associated with prenatal anxiety and depression in pregnant women. Ecotoxicol Environ Saf. (2022) 248:114284. doi: 10.1016/J.ECOENV.2022.114284 36395653

[77] Helbich M Browning MHEM Huss A. Outdoor light at night, air pollution and depressive symptoms: A cross-sectional study in the Netherlands. Sci Total Environ. (2020) 744:140914. doi: 10.1016/J.SCITOTENV.2020.140914 32755781

[78] Münzel T Hahad O Daiber A. The dark side of nocturnal light pollution. Outdoor light at night increases risk of coronary heart disease. Eur Heart J. (2021) 42:831–4. doi: 10.1093/EURHEARTJ/EHAA866 PMC7897459 33221876

[79] Jin J Han W Yang T Xu Z Zhang J Cao R. Artificial light at night, MRI-based measures of brain iron deposition and incidence of multiple mental disorders. Sci Total Environ. (2023) 902:166004. doi: 10.1016/J.SCITOTENV.2023.166004 37544462

[80] Chen Y Tan J Liu Y Dong GH Yang BY Li N. Long-term exposure to outdoor light at night and mild cognitive impairment: A nationwide study in Chinese veterans. Sci Total Environ. (2022) 847:157441. doi: 10.1016/J.SCITOTENV.2022.157441 35863567

[81] Wyse CA Selman C Page MM Coogan AN Hazlerigg DG. Circadian desynchrony and metabolic dysfunction; did light pollution make us fat? Med Hypotheses. (2011) 77:1139–44. doi: 10.1016/J.MEHY.2011.09.023 21983352

[82] Namgyal D Chandan K Sultan A Aftab M Ali S Mehta R. Dim light at night induced neurodegeneration and ameliorative effect of curcumin. Cells. (2020) 9:2093. doi: 10.3390/CELLS9092093 32933226 PMC7565558

[83] Mazzoleni E Vinceti M Costanzini S Garuti C Adani G Vinceti G. Outdoor artificial light at night and risk of early-onset dementia: A case-control study in the Modena population, Northern Italy. Heliyon. (2023) 9:e17837. doi: 10.1016/J.HELIYON.2023.E17837 37455959 PMC10339013

[84] Nash TR Chow ES Law AD Fu SD Fuszara E Bilska A. Daily blue-light exposure shortens lifespan and causes brain neurodegeneration in Drosophila. NPJ Aging Mech Dis. (2019) 5:1–8. doi: 10.1038/s41514-019-0038-6 31636947 PMC6797782

[85] Lucassen EA Coomans CP van Putten M de Kreij SR van Genugten JHLT Sutorius RPM. Environmental 24-hr cycles are essential for health. Curr Biol. (2016) 26:1843–53. doi: 10.1016/J.CUB.2016.05.038 27426518

[86] Menéndez-Velázquez A Morales D García-Delgado AB. Light pollution and circadian misalignment: A healthy, blue-free, white light-emitting diode to avoid chronodisruption. Int J Environ Res Public Health. (2022) 19:1849. doi: 10.3390/IJERPH19031849 35162871 PMC8835293

[87] Uddin MS Sumsuzzman DM Jeandet P Behl T Rauf A Amran MS. Deciphering the interacting mechanisms of circadian disruption and alzheimer’s disease. Neurochem Res. (2021) 46:1603–17. doi: 10.1007/s11064-021-03325-x 33871799

[88] Saeed Y Abbott SM. Circadian disruption associated with alzheimer’s disease. Curr Neurol Neurosci Rep. (2017) 17:29. doi: 10.1007/s11910-017-0745-y 28324298

[89] Wu H Dunnett S Ho YS Chang RCC. The role of sleep deprivation and circadian rhythm disruption as risk factors of Alzheimer’s disease. Front Neuroendocrinol. (2019) 54. doi: 10.1016/J.YFRNE.2019.100764 31102663

[90] Shi L Chen SJ Ma MY Bao YP Han Y Wang YM. Sleep disturbances increase the risk of dementia: A systematic review and meta-analysis. Sleep Med Rev. (2018) 40:4–16. doi: 10.1016/J.SMRV.2017.06.010 28890168

[91] Kang JE Lim MM Bateman RJ Lee JJ Smyth LP Cirrito JR. Amyloid-beta dynamics are regulated by orexin and the sleep-wake cycle. Science. (2009) 326:1005–7. doi: 10.1126/SCIENCE.1180962 PMC2789838 19779148

[92] Li Y Zhang J Wan J Liu A Sun J. Melatonin regulates Aβ production/clearance balance and Aβ neurotoxicity: A potential therapeutic molecule for Alzheimer’s disease. BioMed Pharmacother. (2020) 132:110887. doi: 10.1016/J.BIOPHA.2020.110887 33254429

[93] Slats D Claassen JAHR Verbeek MM Overeem S. Reciprocal interactions between sleep, circadian rhythms and Alzheimer’s disease: focus on the role of hypocretin and melatonin. Ageing Res Rev. (2013) 12:188–200. doi: 10.1016/J.ARR.2012.04.003 22575905

[94] Yulug B Hanoglu L Kilic E. Does sleep disturbance affect the amyloid clearance mechanisms in Alzheimer’s disease? Psychiatry Clin Neurosci. (2017) 71:673–7. doi: 10.1111/PCN.12539 28523718

[95] Karska J Kowalski S Gładka A Brzecka A Sochocka M Kurpas D. Artificial light and neurodegeneration: does light pollution impact the development of Alzheimer’s disease? Geroscience. (2023) 46:87–97. doi: 10.1007/S11357-023-00932-0 PMC10828315 37733222

[96] Moro MF Carta MG Pintus M Pintus E Melis R Kapczinski F. Validation of the italian version of the biological rhythms interview of assessment in neuropsychiatry (BRIAN): some considerations on its screening usefulness. Clin Pract Epidemiol Ment Health. (2014) 10:48–52. doi: 10.2174/1745017901410010048 24987447 PMC4076616

[97] Bedrosian TA Weil ZM Nelson RJ. Chronic dim light at night provokes reversible depression-like phenotype: possible role for TNF. Mol Psychiatry. (2012) 18:930–6. doi: 10.1038/mp.2012.96 22824811

[98] Hidalgo-Mazzei D Reinares M Mateu A Juruena MF Young AH Pérez-Sola V. Is a SIMPLe smartphone application capable of improving biological rhythms in bipolar disorder? J Affect Disord. (2017) 223:10–6. doi: 10.1016/J.JAD.2017.07.028 28711743

[99] Salvadore G Quiroz JA MaChado-Vieira R Henter ID Manji HK Zarate CA. The neurobiology of the switch process in bipolar disorder: a review. J Clin Psychiatry. (2010) 71:1488–501. doi: 10.4088/JCP.09r05259gre PMC3000635 20492846

[100] Raitiere MN. The elusive “Switch process” in bipolar disorder and photoperiodism: A hypothesis centering on NADPH oxidase-generated reactive oxygen species within the bed nucleus of the stria terminalis. Front Psychiatry. (2022) 13:847584. doi: 10.3389/FPSYT.2022.847584 35782417 PMC9243387

[101] Wang HB Tahara Y Luk SHC Kim YS Hitchcock ON MacDowell Kaswan ZA. Melatonin treatment of repetitive behavioral deficits in the Cntnap2 mouse model of autism spectrum disorder. Neurobiol Dis. (2020) 145:105064. doi: 10.1016/J.NBD.2020.105064 32889171 PMC7597927

[102] Paksarian D Rudolph KE Stapp EK Dunster GP He J Mennitt D. Association of outdoor artificial light at night with mental disorders and sleep patterns among US adolescents. JAMA Psychiatry. (2020) 77:1266–75. doi: 10.1001/JAMAPSYCHIATRY.2020.1935 PMC7344797 32639562

[103] Tonon AC Constantino DB Amando GR Abreu AC Francisco AP De Oliveira MAB. Sleep disturbances, circadian activity, and nocturnal light exposure characterize high risk for and current depression in adolescence. Sleep. (2022) 45:zsac104. doi: 10.1093/SLEEP/ZSAC104 35522984

[104] Bedrosian TA Nelson RJ. Timing of light exposure affects mood and brain circuits. Transl Psychiatry. (2017) 7:e1017. doi: 10.1038/TP.2016.262 28140399 PMC5299389

[105] Min Jy Min Kb. Outdoor light at night and the prevalence of depressive symptoms and suicidal behaviors: A cross-sectional study in a nationally representative sample of Korean adults. J Affect Disord. (2018) 227:199–205. doi: 10.1016/J.JAD.2017.10.039 29100153

[106] Chen R Weitzner AS McKennon LA Fonken LK. Light at night during development in mice has modest effects on adulthood behavior and neuroimmune activation. Behav Brain Res. (2021) 405:113171. doi: 10.1016/J.BBR.2021.113171 33577883 PMC8120684

[107] Shanahan L Copeland WE Angold A Bondy CL Costello EJ. Sleep problems predict and are predicted by generalized anxiety/depression and oppositional defiant disorder. J Am Acad Child Adolesc Psychiatry. (2014) 53:550–8. doi: 10.1016/J.JAAC.2013.12.029 PMC4144678 24745954

[108] Schibler U. The daily rhythms of genes, cells and organs. Biological clocks and circadian timing in cells. EMBO Rep. (2005) 6 Spec N:S9–13. doi: 10.1038/SJ.EMBOR.7400424 PMC1369272 15995671

[109] Gachon F Fonjallaz P Damiola F Gos P Kodama T Zakany J. The loss of circadian PAR bZip transcription factors results in epilepsy. Genes Dev. (2004) 18:1397. doi: 10.1101/GAD.301404 15175240 PMC423191

[110] Wang HB Zhou D Luk SHC In Cha H Mac A Chae R. Long wavelength light reduces the negative consequences of dim light at night. Neurobiol Dis. (2023) 176:105944. doi: 10.1016/J.NBD.2022.105944 36493974 PMC10594349

[111] Repova K Baka T Krajcirovicova K Stanko P Aziriova S Reiter RJ. Melatonin as a potential approach to anxiety treatment. Int J Mol Sci. (2022) 23:16187. doi: 10.3390/IJMS232416187 36555831 PMC9788115

[112] Babisch W. Noise and health. Noise Health. (2002) 4:1. doi: 10.1260/1475473021502847 12537836

[113] Manukyan AL. Noise as a cause of neurodegenerative disorders: molecular and cellular mechanisms. Neurol Sci. (2022) 43:2983–93. doi: 10.1007/S10072-022-05948-6 35166975

[114] Münzel T Gori T Babisch W Basner M. Cardiovascular effects of environmental noise exposure. Eur Heart J. (2014) 35:829. doi: 10.1093/EURHEARTJ/EHU030 24616334 PMC3971384

[115] Klompmaker JO Hoek G Bloemsma LD Wijga AH van den Brink C Brunekreef B. Associations of combined exposures to surrounding green, air pollution and traffic noise on mental health. Environ Int. (2019) 129:525–37. doi: 10.1016/J.ENVINT.2019.05.040 31158598

[116] Seidler A Hegewald J Seidler AL Schubert M Wagner M Dröge P. Association between aircraft, road and railway traffic noise and depression in a large case-control study based on secondary data. Environ Res. (2017) 152:263–71. doi: 10.1016/J.ENVRES.2016.10.017 27816007

[117] Min Jy Min Kb. Night noise exposure and risk of death by suicide in adults living in metropolitan areas. Depress Anxiety. (2018) 35:876–83. doi: 10.1002/DA.22789 29953702

[118] Tarnopolsky A Barker SM Wiggins RD McLean EK. The effect of aircraft noise on the mental health of a community sample: a pilot study. Psychol Med. (1978) 8:219–33. doi: 10.1017/S0033291700014276 652896

[119] Hahad O Prochaska JH Daiber A Muenzel T. Environmental noise-induced effects on stress hormones, oxidative stress, and vascular dysfunction: key factors in the relationship between cerebrocardiovascular and psychological disorders. Oxid Med Cell Longev. (2019) 2019:4623109. doi: 10.1155/2019/4623109 31814877 PMC6878772

[120] Truskewycz A Gundry TD Khudur LS Kolobaric A Taha M Aburto-Medina A. Petroleum hydrocarbon contamination in terrestrial ecosystems-fate and microbial responses. Molecules. (2019) 24:3400. doi: 10.3390/MOLECULES24183400 31546774 PMC6767264

[121] Joko T Dewanti NAY Dangiran HL. Pesticide poisoning and the use of personal protective equipment (PPE) in Indonesian farmers. J Environ Public Health. (2020) 2020:5379619. doi: 10.1155/2020/5379619 32405302 PMC7201457

[122] Gan S Lau EV Ng HK. Remediation of soils contaminated with polycyclic aromatic hydrocarbons (PAHs). J Hazard Mater. (2009) 172:532–49. doi: 10.1016/J.JHAZMAT.2009.07.118 19700241

[123] Obmiński A. Asbestos cement products and their impact on soil contamination in relation to various sources of anthropogenic and natural asbestos pollution. Sci Total Environ. (2022) 848:157275. doi: 10.1016/J.SCITOTENV.2022.157275 35905955

[124] Neal AP Worley PF Guilarte TR. Lead exposure during synaptogenesis alters NMDA receptor targeting via NMDA receptor inhibition. Neurotoxicology. (2011) 32:281–9. doi: 10.1016/J.NEURO.2010.12.013 PMC3049857 21192972

[125] Modabbernia A Arora M Reichenberg A. Environmental exposure to metals, neurodevelopment, and psychosis. Curr Opin Pediatr. (2016) 28:243–9. doi: 10.1097/MOP.0000000000000332 26867166

[126] Ayuso-Álvarez A Simón L Nuñez O Rodríguez-Blázquez C Martín-Méndez I Bel-lán A. Association between heavy metals and metalloids in topsoil and mental health in the adult population of Spain. Environ Res. (2019) 179:108784. doi: 10.1016/J.ENVRES.2019.108784 31606614

[127] Vandermoere F. Psychosocial health of residents exposed to soil pollution in a Flemish neighbourhood. Soc Sci Med. (2008) 66:1646–57. doi: 10.1016/J.SOCSCIMED.2007.12.031 18237836

[128] Rezaei Kalantary R Jaffarzadeh N Rezapour M Hesami Arani M. Association between exposure to polycyclic aromatic hydrocarbons and attention deficit hyperactivity disorder in children: a systematic review and meta-analysis. Environ Sci pollut Res Int. (2020) 27:11531–40. doi: 10.1007/S11356-020-08134-3 32124297

[129] Clemente M Reig-Botella A Prados JC. Alterations in psychosocial health of people affected by asbestos poisoning. Rev Saude Publica. (2015) 49:24. doi: 10.1590/S0034-8910.2015049005445 25902564 PMC4390070

[130] Campos É dos Santos Pinto da Silva V Sarpa Campos de Mello M Barros Otero U. Exposure to pesticides and mental disorders in a rural population of Southern Brazil. Neurotoxicology. (2016) 56:7–16. doi: 10.1016/J.NEURO.2016.06.002 27350176

[131] Ong-Artborirak P Boonchieng W Juntarawijit Y Juntarawijit C. Potential effects on mental health status associated with occupational exposure to pesticides among thai farmers. Int J Environ Res Public Health. (2022) 19:9654. doi: 10.3390/IJERPH19159654 35955007 PMC9367823

[132] Cherry N Burstyn I Beach J Senthilselvan A. Mental health in Alberta grain farmers using pesticides over many years. Occup Med (Lond). (2012) 62:400–6. doi: 10.1093/OCCMED/KQS136 22915560

[133] Von Ehrenstein OS Ling C Cui X Cockburn M Park AS Yu F. Prenatal and infant exposure to ambient pesticides and autism spectrum disorder in children: population based case-control study. BMJ. (2019) 364:l962. doi: 10.1136/BMJ.L962 30894343 PMC6425996

[134] Serrano-Medina A Ugalde-Lizárraga A Bojorquez-Cuevas MS Garnica-Ruiz J González-Corral MA García-Ledezma A. Neuropsychiatric disorders in farmers associated with organophosphorus pesticide exposure in a rural village of northwest méxico. Int J Environ Res Public Health. (2019) 16:689. doi: 10.3390/IJERPH16050689 30813607 PMC6427808

[135] Hurley RA Taber KH. Occupational exposure to solvents: neuropsychiatric and imaging features. J Neuropsychiatry Clin Neurosci. (2015) 27:1–7. doi: 10.1176/APPI.NEUROPSYCH.270101 25716516

[136] Wang Q Yang Z. Industrial water pollution, water environment treatment, and health risks in China. Environ pollut. (2016) 218:358–65. doi: 10.1016/J.ENVPOL.2016.07.011 27443951

[137] O’Neill MS Jerrett M Kawachi I Levy JI Cohen AJ Gouveia N. Health, wealth, and air pollution: advancing theory and methods. Environ Health Perspect. (2003) 111:1861–70. doi: 10.1289/EHP.6334 PMC1241758 14644658

[138] Rehman K Fatima F Waheed I Akash MSH. Prevalence of exposure of heavy metals and their impact on health consequences. J Cell Biochem. (2018) 119:157–84. doi: 10.1002/JCB.26234 28643849

[139] Goodlad JK Marcus DK Fulton JJ. Lead and Attention-Deficit/Hyperactivity Disorder (ADHD) symptoms: a meta-analysis. Clin Psychol Rev. (2013) 33:417–25. doi: 10.1016/J.CPR.2013.01.009 23419800

[140] Kim S Arora M Fernandez C Landero J Caruso J Chen A. Lead, mercury, and cadmium exposure and attention deficit hyperactivity disorder in children. Environ Res. (2013) 126:105–10. doi: 10.1016/J.ENVRES.2013.08.008 PMC3847899 24034783

[141] Neugebauer J Wittsiepe J Kasper-Sonnenberg M Schöneck N Schölmerich A Wilhelm M. The influence of low level pre- and perinatal exposure to PCDD/Fs, PCBs, and lead on attention performance and attention-related behavior among German school-aged children: results from the Duisburg Birth Cohort Study. Int J Hyg Environ Health. (2015) 218:153–62. doi: 10.1016/J.IJHEH.2014.09.005 25456149

[142] Brinkel J Khan MH Kraemer A. A systematic review of arsenic exposure and its social and mental health effects with special reference to Bangladesh. Int J Environ Res Public Health. (2009) 6:1609–19. doi: 10.3390/IJERPH6051609 PMC2697931 19543409

[143] Fujino Y Guo X Liu J You L Miyatake M Yoshimura T. Mental health burden amongst inhabitants of an arsenic-affected area in Inner Mongolia, China. Soc Sci Med. (2004) 59:1969–73. doi: 10.1016/J.SOCSCIMED.2004.02.031 15312930

[144] Zierold KM Knobeloch L Anderson H. Prevalence of chronic diseases in adults exposed to arsenic-contaminated drinking water. Am J Public Health. (2004) 94:1936. doi: 10.2105/AJPH.94.11.1936 15514231 PMC1448563

[145] Rihmer Z Hal M Kapitány B Gonda X Vargha M Döme P. Preliminary investigation of the possible association between arsenic levels in drinking water and suicide mortality. J Affect Disord. (2015) 182:23–5. doi: 10.1016/J.JAD.2015.04.034 25965691

[146] Skogheim TS Weyde KVF Engel SM Aase H Surén P Øie MG. Metal and essential element concentrations during pregnancy and associations with autism spectrum disorder and attention-deficit/hyperactivity disorder in children. Environ Int. (2021) 152:106468. doi: 10.1016/J.ENVINT.2021.106468 33765546

[147] Forns J Fort M Casas M Cáceres A Guxens M Gascon M. Exposure to metals during pregnancy and neuropsychological development at the age of 4 years. Neurotoxicology. (2014) 40:16–22. doi: 10.1016/J.NEURO.2013.10.006 24211492

[148] Long M Ghisari M Kjeldsen L Wielsøe M Nørgaard-Pedersen B Mortensen EL. Autism spectrum disorders, endocrine disrupting compounds, and heavy metals in amniotic fluid: A case-control study. Mol Autism. (2019) 10:1–19. doi: 10.1186/s13229-018-0253-1 30647876 PMC6327542

[149] Aschengrau A Weinberg JM Janulewicz PA Romano ME Gallagher LG Winter MR. Occurrence of mental illness following prenatal and early childhood exposure to tetrachloroethylene (PCE)-contaminated drinking water: A retrospective cohort study. Environ Health. (2012) 11:1–12. doi: 10.1186/1476-069X-11-2/TABLES/3 22264316 PMC3292942

[150] Lv J Zhang L Chen Y Ye B Han J Jin N. Occurrence and distribution of pharmaceuticals in raw, finished, and drinking water from seven large river basins in China. J Water Health. (2019) 17:477–89. doi: 10.2166/WH.2019.250 31095522

[151] Kaushik G Thomas MA. The potential association of psychoactive pharmaceuticals in the environment with human neurological disorders. Sustain Chem Pharm. (2019) 13:100148. doi: 10.1016/J.SCP.2019.100148 31453309 PMC6709680

[152] Goldstein BD Osofsky HJ Lichtveld MY. The gulf oil spill. New Engl J Med. (2011) 364:1334–48. doi: 10.1056/NEJMRA1007197 21470011

[153] Arata CM Picou JS Johnson GD McNally TS. Coping with technological disaster: an application of the conservation of resources model to the Exxon Valdez oil spill. J Trauma Stress. (2000) 13:23–39. doi: 10.1023/A:1007764729337 10761172

[154] Cuthbertson CA Newkirk C Ilardo J Loveridge S Skidmore M. Angry, scared, and unsure: mental health consequences of contaminated water in flint, michigan. J Urban Health. (2016) 93:899–908. doi: 10.1007/s11524-016-0089-y 27807700 PMC5126025

[155] Carrasco JM Pérez-Gómez B García-Mendizábal MJ Lope V Aragonés N Forjaz MJ. Health-related quality of life and mental health in the medium-term aftermath of the Prestige oil spill in Galiza (Spain): a cross-sectional study. BMC Public Health. (2007) 7:245. doi: 10.1186/1471-2458-7-245 17875207 PMC2194772

[156] Hrabok M Delorme A Agyapong VIO. Threats to mental health and well-being associated with climate change. J Anxiety Disord. (2020) 76:102295. doi: 10.1016/J.JANXDIS.2020.102295 32896782

[157] Barkin JL Buoli M Curry CL von Esenwein SA Upadhyay S Kearney MB. Effects of extreme weather events on child mood and behavior. Dev Med Child Neurol. (2021) 63:785–90. doi: 10.1111/DMCN.14856 PMC8252647 33720406

[158] Clayton S. Climate change and mental health. Curr Environ Health Rep. (2021) 8:1–6. doi: 10.1007/s40572-020-00303-3 33389625

[159] Lowe SR Bonumwezi JL Valdespino-Hayden Z Galea S. Posttraumatic stress and depression in the aftermath of environmental disasters: A review of quantitative studies published in 2018. Curr Environ Health Rep. (2019) 6:344–60. doi: 10.1007/S40572-019-00245-5 31487033

[160] Garfin DR Thompson RR Holman EA Wong-Parodi G Silver RC. Association between repeated exposure to hurricanes and mental health in a representative sample of florida residents. JAMA Netw Open. (2022) 5:E2217251. doi: 10.1001/JAMANETWORKOPEN.2022.17251 35708689 PMC9204543

[161] Nakabayashi M. Cumulative adverse mental health outcomes after concurrent disasters-social, scientific, and policy-making implications. JAMA Netw Open. (2022) 5:E2217260. doi: 10.1001/JAMANETWORKOPEN.2022.17260 35708693

[162] López-Cepero A O’Neill HJ Marrero A Falcon LM Tamez M Rodríguez-Orengo JF. Association between adverse experiences during Hurricane María and mental and emotional distress among adults in Puerto Rico. Soc Psychiatry Psychiatr Epidemiol. (2022) 57:2423–32. doi: 10.1007/S00127-022-02355-2 PMC9434507 36048184

[163] Schwartz RM Gillezeau CN Liu B Lieberman-Cribbin W Taioli E. Longitudinal impact of hurricane sandy exposure on mental health symptoms. Int J Environ Res Public Health. (2017) 14:957. doi: 10.3390/IJERPH14090957 28837111 PMC5615494

[164] Raker EJ Lowe SR Arcaya MC Johnson ST Rhodes J Waters MC. Twelve years later: The long-term mental health consequences of Hurricane Katrina. Soc Sci Med. (2019) 242:112610. doi: 10.1016/J.SOCSCIMED.2019.112610 31677480 PMC8450020

[165] Chique C Hynds P Nyhan MM Lambert S Boudou M O’Dwyer J. Psychological impairment and extreme weather event (EWE) exposure, 1980-2020: A global pooled analysis integrating mental health and well-being metrics. Int J Hyg Environ Health. (2021) 238:113840. doi: 10.1016/J.IJHEH.2021.113840 34543982

[166] Chen L Liu A. The incidence of posttraumatic stress disorder after floods: A meta-analysis. Disaster Med Public Health Prep. (2015) 9:329–33. doi: 10.1017/DMP.2015.17 25857395

[167] Weilnhammer V Schmid J Mittermeier I Schreiber F Jiang L Pastuhovic V. Extreme weather events in europe and their health consequences - A systematic review. Int J Hyg Environ Health. (2021) 233:113688. doi: 10.1016/J.IJHEH.2021.113688 33530011

[168] Bandla S Nappinnai NR Gopalasamy S. Psychiatric morbidity in December 2015 flood-affected population in Tamil Nadu, India. Int J Soc Psychiatry. (2019) 65:338–44. doi: 10.1177/0020764019846166 31068043

[169] Charlson F Ali S Benmarhnia T Pearl M Massazza A Augustinavicius J. Climate change and mental health: A scoping review. Int J Environ Res Public Health. (2021) 18:4486. doi: 10.3390/IJERPH18094486 33922573 PMC8122895

[170] Mirabelli MC Vaidyanathan A Pennington AF Ye D Trenga CA. Wildfire smoke and symptoms affecting mental health among adults in the U.S. state of Oregon. Prev Med (Baltim). (2022) 164:107333. doi: 10.1016/J.YPMED.2022.107333 PMC9691586 36336164

[171] Silveira S Kornbluh M Withers MC Grennan G Ramanathan V Mishra J. Chronic mental health sequelae of climate change extremes: A case study of the deadliest Californian wildfire. Int J Environ Res Public Health. (2021) 18:1–15. doi: 10.3390/IJERPH18041487 PMC7915298 33557397

[172] Zhang Y Workman A Russell MA Williamson M Pan H Reifels L. The long-term impact of bushfires on the mental health of Australians: a systematic review and meta-analysis. Eur J Psychotraumatol. (2022) 13:1–15. doi: 10.1080/20008198.2022.2087980 PMC9359172 35957633

[173] Hanigan IC Butler CD Kokic PN Hutchinson MF. Suicide and drought in new south wales, Australia, 1970-2007. Proc Natl Acad Sci U.S.A. (2012) 109:13950–5. doi: 10.1073/PNAS.1112965109 PMC3435226 22891347

[174] Hanigan IC Schirmer J Niyonsenga T. Drought and distress in southeastern Australia. Ecohealth. (2018) 15:642–55. doi: 10.1007/S10393-018-1339-0 29797158

[175] OBrien LV Berry HL Coleman C Hanigan IC. Drought as a mental health exposure. Environ Res. (2014) 131:181–7. doi: 10.1016/J.ENVRES.2014.03.014 24727641

[176] Thoma MV Rohleder N Rohner SL. Clinical ecopsychology: the mental health impacts and underlying pathways of the climate and environmental crisis. Front Psychiatry. (2021) 12:675936. doi: 10.3389/FPSYT.2021.675936 34093283 PMC8175799

[177] Hong JS Hyun SY Lee JH Sim M. Mental health effects of the Gangwon wildfires. BMC Public Health. (2022) 22:1183. doi: 10.1186/S12889-022-13560-8 35701801 PMC9195206

[178] Graham H White P Cotton J McManus S. Flood- and weather-damaged homes and mental health: an analysis using England’s mental health survey. Int J Environ Res Public Health. (2019) 16:3256. doi: 10.3390/IJERPH16183256 31491859 PMC6765946

[179] Luong TT Handley T Austin EK Kiem AS Rich JL Kelly B. New insights into the relationship between drought and mental health emerging from the Australian rural mental health study. Front Psychiatry. (2021) 12:719786. doi: 10.3389/FPSYT.2021.719786 34539467 PMC8440818

[180] Edwards B Gray M Hunter B. The impact of drought on mental health in rural and regional Australia. Soc Indic Res. (2015) 121:177–94. doi: 10.1007/S11205-014-0638-2

